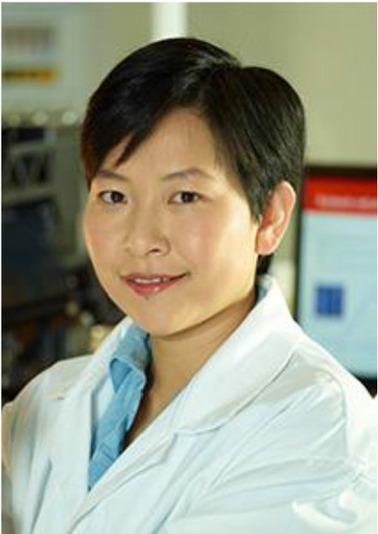# Solar Energy Catalysis

**DOI:** 10.1002/anie.202204880

**Published:** 2022-05-31

**Authors:** Xiaodong Sun, Shuaiyu Jiang, Hongwei Huang, Hui Li, Baohua Jia, Tianyi Ma

**Affiliations:** ^1^ Institute of Clean Energy Chemistry Key Laboratory for Green Synthesis and Preparative Chemistry of Advanced Materials College of Chemistry Liaoning University Shenyang 110036 P. R. China; ^2^ School of Science RMIT University Melbourne VIC 3000 Australia; ^3^ Beijing Key Laboratory of Materials Utilization of Nonmetallic Minerals and Solid Wastes National Laboratory of Mineral Materials School of Materials Science and Technology China University of Geosciences Beijing 100083 China

**Keywords:** Photocatalysis, Photothermal Catalysis, Pyroelectric Catalysis, Solar Cell Powered Electrocatalysis, Solar Energy

## Abstract

When it comes to using solar energy to promote catalytic reactions, photocatalysis technology is the first choice. However, sunlight can not only be directly converted into chemical energy through a photocatalytic process, it can also be converted through different energy‐transfer pathways. Using sunlight as the energy source, photocatalytic reactions can proceed independently, and can also be coupled with other catalytic technologies to enhance the overall catalytic efficiency. Therefore, sunlight‐driven catalytic reactions are diverse, and need to be given a specific definition. We propose a timely perspective for catalytic reactions driven by sunlight and give them a specific definition, namely “solar energy catalysis”. The concept of different types of solar energy catalysis, such as photocatalysis, photothermal catalysis, solar cell powered electrocatalysis, and pyroelectric catalysis, are highlighted. Finally, their limitations and future research directions are discussed.

## Graphical abstract

Solar energy catalysis is a specific and “all‐in‐one” definition for catalytic reactions that utilize solar light as the energy input. Based on the different conversion pathways, solar energy catalysis can be divided into photocatalysis, photothermal catalysis, solar cell powered catalysis, pyroelectric catalysis, and the combined effect of some of these processes.

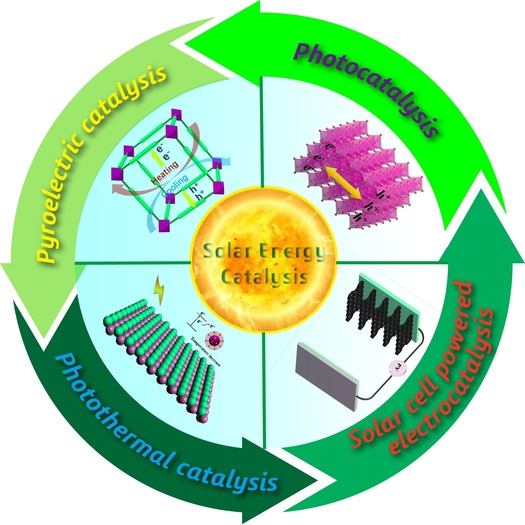

## 1 Introduction

With the rapid development of technology, demands for energy sources continue to increase. The depletion of traditional fossil fuels not only causes energy shortages but also results in many issues regarding environmental pollution, which are the main obstacles to the development of a sustainable society.^[1]^ Therefore, clean and renewable energy is urgently required. By virtue of its abundance and sustainability, solar energy has been recognized as the most promising energy source, and it will play the most important role in the energy‐source structure of the future. In 1972, Fujishima and Honda first reported that TiO_2_ can be used as a photoelectrode for water splitting. Since then, photocatalysis, a technology that can directly convert solar energy into chemical energy, has attracted tremendous interest and has been considered as one of the most promising technologies to solve the energy and environmental issues.^[2]^

Recently, with in‐depth research on solar energy, researchers have gradually recognized that solar energy can not only be directly transformed into chemical energy through photocatalytic processes, but can also be transformed into thermal energy and electrical energy to initiate different kinds of catalytic reactions, including thermal catalysis,^[3]^ pyroelectric catalysis,^[4]^ and electrocatalysis.^[5]^ To further enhance the efficiency of solar energy utilization, strategies for designing coupled catalytic systems that can combine the advantages of different types of catalysis have also aroused broad interest and have been widely studied in recent years. For example, poor solar utilization efficiency and low apparent quantum efficiencies (AQEs) are major obstacles for photocatalysis, since most of the efficient photocatalysts only display a strong response in the ultraviolet (UV) region and have a lack of light–matter interactions. However, plasmonic metals can make full use of the sunlight in the visible and infrared (IR) regions by not only inducing local thermal effects and converting light energy into thermal energy, but also by generating hot carriers.^[6]^ Therefore, by combining the advantages of photocatalysis and thermal catalysis, reactions can be promoted by synergy effects. Although catalytic reactions which adopt solar energy as the energy source are attractive, the species involved in them are diverse and have never been summarized and classified systematically. Therefore, there is an urgent need for a timely scientific perspective for such a kind of catalytic reaction as well as a specific definition to avoid confusion.

This Minireview aims to give constructive insight into sunlight‐triggered reactions and give them a specific and “all‐in‐one” definition, namely, “Solar Energy Catalysis” (the SEC process). In this Minireview, we divide the SEC process into different categories based on the energy‐transfer pathways, namely catalytic technologies which can convert solar energy directly or indirectly into chemical energy (photocatalysis and photocatalysis with an up‐conversion effect), convert solar energy into thermal energy and then to chemical energy (photothermal catalysis and pyroelectric catalysis), and convert solar energy into electrical energy and then to chemical energy (solar cell promoted catalysis). Finally, limitations and the future research direction of solar energy catalysis are discussed. This Minireview aims to provide constructive insights and advice on the concept of solar energy catalysis.

## 2 Concept and Categories of Solar Energy Catalysis

Solar energy catalysis is a specific and “all‐in‐one” definition for the kind of catalytic reactions that utilize solar light as the energy input. Solar energy catalysis can be divided into photocatalysis, photocatalysis promoted by the up‐conversion effect, photothermal catalysis, solar cell powered catalysis, and pyroelectric catalysis, depending on the energy conversion pathway.

### 2.1 Photocatalysis and Up‐Conversion Effect Promoted Photocatalysis

Photocatalysis is a green technology that can directly convert renewable solar energy into chemical energy. By utilizing solar energy as the driving force, various reactions can be initiated, such as water splitting,^[7]^ CO_2_ reduction,^[8]^ N_2_ reduction,^[9]^ organic synthesis,^[10]^ cancer therapy,^[11]^ self‐cleaning as well as elimination of pollutants.^[12]^ In the photocatalytic system, semiconductors with a band gap (*E*_g_) between 0.1 eV and 4 eV are always used as photocatalysts. Exposure of the semiconductors to sunlight or an illuminated light source can result in the light energy being absorbed. When the photon energy is higher than their band gap energy, the semiconductors can be excited, thereby generating electron–hole pairs, from which the electrons migrate to the conduction band (CB) and leave the holes in the valence band (VB). However, by virtue of the low charge‐transfer ability, photogenerated electrons and holes will recombine during this process and seriously influence the light utilization efficiency of the photocatalysts. Then, the electron hole without recombination will immediately migrate to the surface reactive sites on the semiconductors. Lastly, the electrons and holes will react with the substrates to trigger the reduction and oxidation reactions, respectively. So far, photocatalysis has been widely studied, and many reviews about this technology have been reported. In this section, we will take the overall splitting of water and reduction of CO_2_ as examples to introduce photocatalysis. The overall splitting of water is a thermodynamically uphill reaction, which needs enough energy to be imported to overcome the energy barrier (Δ*G* ^0^=237 kJ mol^−1^). The reaction mechanism can be described by Equations (1)–3:

(1) $$2\;H{}^{+}+2\;e{}^{-}\to H{}_{2}\;\left(\mathrm{reduction}\;\mathrm{half}\text{-}\mathrm{reaction}\right)$$

(2) $$2\;H{}_{2}O+2\;h{}^{+}\to 4\;H{}^{+}+O{}_{2}\;\left(\mathrm{oxidation}\;\mathrm{half}\text{-}\mathrm{reaction}\right)$$

(3) $$H{}_{2}O\to H{}_{2}+1/2\;O{}_{2}\;\left(\mathrm{overall}\;\mathrm{water}\;\mathrm{splitting}\right)$$

After irradiation with light, the electrons and holes will be generated and transported to the surface of the photocatalyst to participate in the redox reaction of water splitting. The electrons in the CB will react with the H^+^ ions to produce H_2_; simultaneously, the holes in the VB will oxidize the water to produce O_2_, thus completing the overall splitting of water. The following two points need to be satisfied at the same time for the water splitting reaction to occur: First, the band gap of the semiconductors should be larger than 1.23 eV. Second, the CB position of the semiconductor should be more negative than 0 V (*E*(H^+^/H_2_)), and correspondingly the VB position should be more positive than +1.23 V (*E*(H_2_O/O_2_).^[13]^ Apart from the above factors, the fast recombination rate of the photogenerated electron pairs, limited light harvesting performance, and insufficient surface reactive sites also restrict the semiconductor from completing the overall water splitting reaction. Only a few semiconductors have been reported to be able to achieve overall water splitting through one‐step excitation. Benefitting from its suitable band structure and excellent UV light absorption ability, SrTiO_3_ has been demonstrated to be an ideal semiconductor to trigger the overall splitting of water.^[14]^ For example, Li and co‐workers successfully constructed an anisotropic SrTiO_3_ with 18 facets by using a strategy to tailor the nanocrystal morphology. In comparison with the isotropic SrTiO_3_ with 6 facets, the electron–hole pairs in the 18‐facet SrTiO_3_ can be more effectively separated on different facets because of the facet anisotropicity. Therefore, the reduction and oxidation reactions can occur on separated facets with an improved overall water‐splitting performance.^[15]^ Although some semiconductors have been indicated to be able to achieve overall water splitting,^[16]^ the severe main problem of the recombination of electron–hole pairs greatly reduced their photocatalytic performance. In this regard, constructing Z‐scheme composites with two‐stage excitation systems has been regarded as the most efficient way to complete the reaction of H_2_ and O_2_ evolution.^[17]^ The Z‐scheme photocatalytic water‐splitting system is composed of H_2_ evolution catalysts, O_2_ evolution catalysts, and an electron conductor or redox mediator. During the photocatalytic process, the electrons with a lower CB position in the O_2_ evolution catalysts will recombine with the holes with a higher VB position in the H_2_ evolution catalysts through an electron conductor or redox mediator. Thus, the leaving electrons with a higher CB position (stronger reduction ability) in the H_2_ evolution catalysts can react with H^+^ ions to produce H_2_, and the leaving holes with a lower VB position (stronger oxidation ability) in the O_2_ evolution catalysts will trigger the reaction of H_2_O oxidation to produce O_2_. Therefore, the Z‐scheme photocatalytic systems can not only improve the electron–hole pairs separation performance, but can also preserve the high redox ability of the composite materials. For example, a Z‐scheme photocatalytic system was successfully designed through integrating the structured single layer perovskite Bi_4_NbO_8_Cl with Rh‐doped SrTiO_3_. Benefitting from the presence of a heterojunction, the separation/migration ability of the charge carriers can be effectively separated. Therefore, with Fe^3+^/Fe^2+^ as a redox mediator, the photocatalytic evolution of H_2_ and O_2_ can be efficiently performed on SrTiO_3_ and Bi_4_NbO_8_Cl, respectively. Although, the photocatalytic water‐splitting technology is attractive, the reported highest solar‐to‐hydrogen (STH) energy conversion efficiency (approximately 1 %) is still far from industrial requirements (5–10 %).^[17a]^

Apart for photocatalytic water splitting, the photocatalytic CO_2_ reduction is also a thermodynamically uphill reaction, which has attracted tremendous interest from academia and industry. According to the different numbers of electrons transferred, the main products of the CO_2_ reduction are multiple, including CO, HCOOH, CH_4_, C_2_H_5_OH, etc. However, by virtue of the high dissociation energy of the C=O bond (750 kJ mol^−1^), CO_2_ molecules possess extraordinary thermodynamic stability, thus greatly increasing the difficulty in triggering the CO_2_ reduction. Moreover, the CO_2_ reduction reaction is kinetically challenging, and involves multiple electron‐transfer processes.^[8]^ Therefore, there is an urgent need to develop highly efficient photocatalysts to solve the above issues. There are five main points to guide the design of catalysts with high photocatalytic activity for CO_2_ reduction: 1) the CB position of photocatalysts should satisfy the thermodynamic requirement of CO_2_ reduction; 2) the photocatalysts should display excellent light‐harvesting performances to produce more carriers; 3) the separation/migration ability of the electron–hole pairs of the photocatalysts should be excellent; 4) the photocatalysts should provide abundant reactive sites to activate the CO_2_ molecules, thus reducing the activation energy as much as possible; and 5) the products should be easily desorbed from the photocatalysts and the VB position of the photocatalysts should satisfy the thermodynamic requirements of H_2_O oxidation. One fascinating example was reported by the Lan group. They proposed a universal strategy to construct a Z‐scheme photocatalytic system with a series of connected covalent bonds by combining covalent organic frameworks materials for CO_2_ reduction and water‐oxidation semiconductors (TiO_2_, Bi_2_WO_6_, and α‐Fe_2_O_3_). In this Z‐scheme photocatalytic system, the cyano/pyridine moieties can serve as the active sites to activate CO_2_ molecules and accumulate electrons to promote CO_2_ reduction, and the holes in the semiconductors can promote the water oxidation. The electrons in the semiconductors and holes in the COFs can recombine at their contact interface through covalent bonds, which is beneficial for the separation of electron–hole pairs. Therefore, the resulting photocatalysts displayed remarkable performance for converting CO_2_ into CO without additional photosensitizers or sacrificial agents.^[8d]^

**Figure 1:**
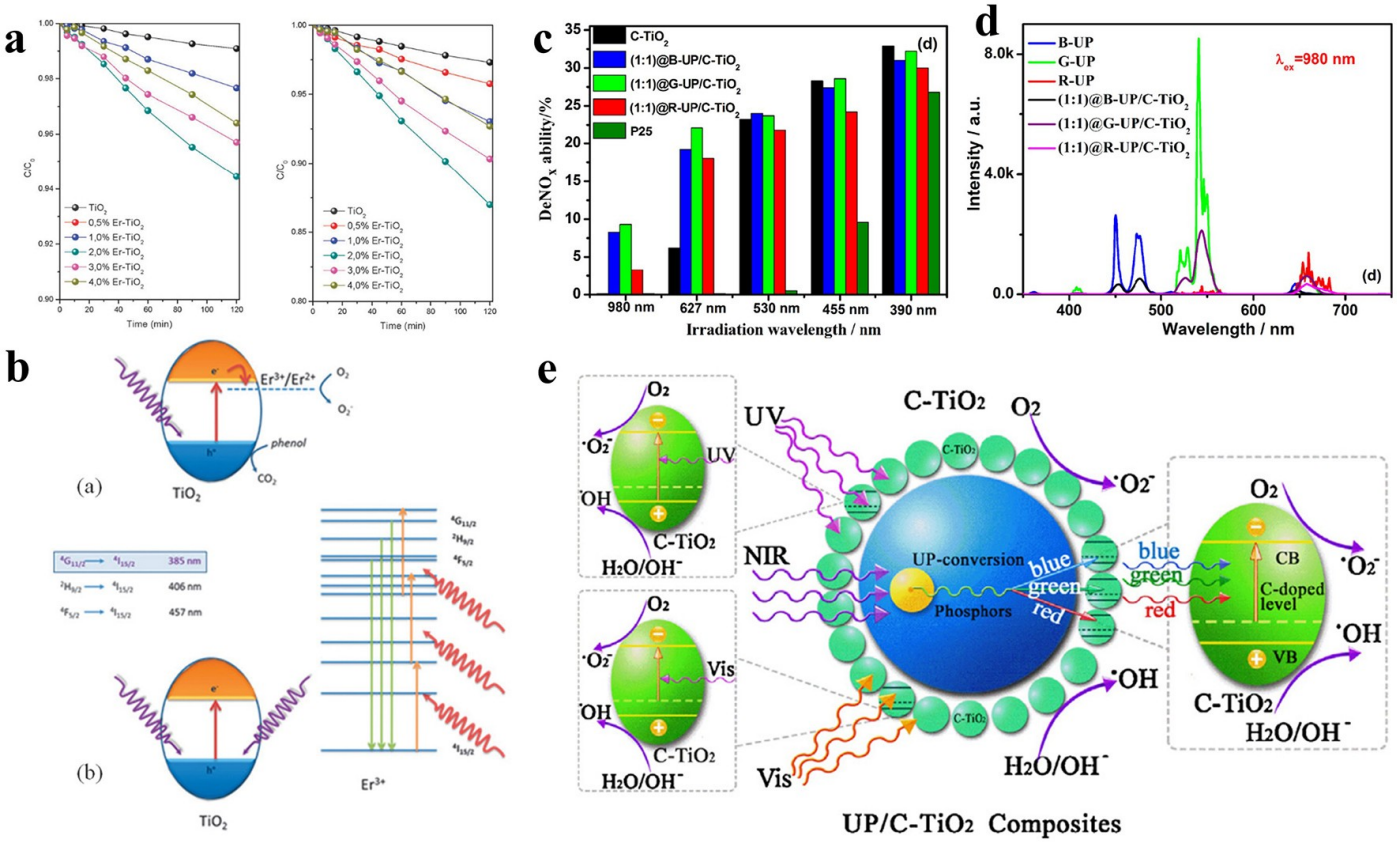
a) Conversion plots for Er^3+^−TiO_2_ systems under excitation with Vis/NIR light for the degradation of phenol (left) and methylene blue (right). b) Mechanism of the photocatalytic induction process in the Er^3+^−TiO_2_ heterostructures: Top: UV‐assisted mechanism and bottom: NIR up‐conversion assisted mechanism. Reproduced with permission.^[18a]^ Copyright 2012, Royal Society of Chemistry. c) NO*x* destruction activities of C−TiO_2_, B−UP/C−TiO_2_, G−UP/C−TiO_2_, R−UP/C−TiO_2_, and P25 under irradiation with light of different wavelengths. d) PL spectra of B−UP, G−UP, R−UP, and three composites upon excitation at 980 nm. e) Mechanism of UV, visible and NIR lights induced photocatalysis of up‐conversion phosphors coupled C‐TiO_2_ composites. Reproduced with permission.^[18c]^ Copyright 2014, Elsevier.

Although the photocatalysis technology is attractive, most semiconductors can only harvest UV light or a small part of visible light, thus resulting in poor light harvesting with a low apparent quantum efficiency (AQE). Therefore, widening the light absorption region of the photocatalysts is regarded as an effective way to promote their photocatalytic performance. In this regard, various strategies, such as elemental doping, defect engineering, surface modification, photosensitive molecular coating, as well as heterojunction constructions, have been proposed to address the above issues. However, the improvement in the light absorption performance of the photocatalysts is extremely limited, with most strategies only able to extend the light harvesting to the visible light region. Thus, exploring more effective methods to widen the light absorption region of the photocatalysts—not limited to visible light, but even to near‐infrared (NIR) or IR regions—is necessary. The intrinsic properties of the photocatalysts are hard to change, but if the incident light can be changed from IR or NIR to the UV or visible region, and thus increase the photon energy during the photocatalytic process, the IR or NIR light, which accounts for about 49 % of the solar spectrum, can be fully used. Inspired by the above, researchers found that introducing up‐conversion technology into photocatalysis is a judicious way to achieve the above hypothesis. The up‐conversion effect, which can also be called the anti‐stokes effect, means that when the materials are excited by low‐energy light, the light can be converted to emit high‐energy light, that is, change light with a long wavelength and low frequency to light with a short wavelength and high frequency.^[18]^ In this regard, through an indirect energy conversion process, the IR or NIR light can be harvested by the photocatalysts. Introducing transition‐metal, lanthanide, or actinide ions into the photocatalysts is an efficient way for endowing them with up‐conversion effects. For example, through a surfactant‐free hydrothermal strategy, Colón and co‐workers successfully introduced Er^3+^ ions into the TiO_2_ matrix to construct Er^3+^−TiO_2_ photocatalysts (Figure 1a, b). The presence of Er^3+^ ions can have a great effect on enhancing the photoactivity of the TiO_2_ for phenol degradation in regard to two aspects. First, the Er^3+^ ions can act as trapping sites for the electrons, thus the reduced Er^2+^ ions can participate in the O_2_ reduction reaction and generate the O_2_^−^ active species. Therefore, the recombination of the electron–hole pairs will be prevented. More interestingly, the Er^3+^ ions can also induce an up‐conversion effect, in which the NIR light can be transformed to UV and visible light through a multistep excitation process. In this case, the light‐absorption performance of the TiO_2_ will be extended to the NIR region.^[18a]^ However, because of the low intensity of Ln^3+^ emissions, the up‐conversion effect is limited and not obvious. In this case, combining photocatalysts with some typical up‐conversion materials, such as lanthanide‐doped NaYF_4_, YF_3_, Y_2_O_3_, carbon, vanadate, and molybdenum disulfide quantum dots, as well as YOF, to fabricate the composite will be an efficient way to enhance the up‐conversion effect. In this composite, the NIR light cannot be directly utilized by the photocatalyst, and the up‐conversion materials will first transform the low‐energy light (IR or NIR light) to high‐energy light (UV or visible light). Then, the transformed light can be reharvested by the photocatalysts. One typical example of photocatalysis promoted by an up‐conversion effect was reported by the Yin group. Considering that up‐conversion phosphors with different emission colors have a significant impact on the photoluminescence properties, the authors combined the C−TiO_2_ with three different phosphors that emitted different up‐conversion emission colors (blue, green, red) to successfully fabricate three different up‐conversion phosphors coupled C−TiO_2_ composites, named B−UP/C−TiO_2_, G−UP/C−TiO_2_, and R−UP/C−TiO_2_. All the composites displayed a higher photocatalytic activity than pure P25, especially G−UP/C−TiO_2_, because, in comparison with B−UP and R−UP, the G−UP material can more effectively convert NIR light with its more‐green photons. Through a series of spectral and photocatalytic tests, the improved photocatalytic performance was attributed to the introduction of up‐conversion phosphors that enable the NIR light to be up‐converted to light of different short wavelengths through a multiphoton mechanism. In this case, the up‐converted light can be absorbed, thus exciting the C−TiO_2_. As for C−TiO_2_, the phosphors can be excited by UV and visible light. In consequence, the up‐conversion phosphor‐coupled C−TiO_2_ composites displayed a large response to UV, visible, and NIR light, as shown by the high photocatalytic activities for the degradation of RhB and destruction of NO gas (Figure 1c, e).^[18c]^

### 2.2 Photothermal Catalysis

Apart from the direct pathway of solar energy to chemical energy, solar energy can also be transformed to thermal energy through the photothermal effect, and thereby promoting the catalytic reactions. The photothermal effect has three different mechanisms: nonradiative relaxation, thermal vibration, and plasmonic heating. The electron–hole pairs will be produced on a semiconductor when the energy of the photons is higher than the band gaps under irradiation with light. Then, only the electron–hole pairs whose location is higher than the band gap can relax to the band edges through nonradiative relaxation, and finally the thermal energy will be produced through the relaxation of excess energy. The thermal vibration mechanism is similar to the nonradiative relaxation mechanism, but it is suitable for organic molecules, such as carbonaceous and some polymeric materials with abundant conjugated π bonds. Under electromagnetic irradiation, a collective oscillation of electrons will be induced at the metal/dielectric or metal/vacuum interface and result in localized surface plasmon resonance (LSPR) if the oscillation frequency matches well with the frequency of the incident photons. Under the LSPR excitation, the plasmonic metals will interact with incident photons and exhibit unique optical responses, which can effectively transform the light energy into heat and energetic hot carriers.^[19]^ LSPR can be observed in some metals, such as Au, Ag, Cu, as well as Al, with high electron mobility. Moreover, the optical performance of the plasmonic metals can also be tuned through regulation of the nanostructures. For example, through increasing the particle size of the Au nanoparticles from 9 nm to 99 nm, their plasmonic absorption will display a red‐shift. Their LSPR spectra can also be regulated by regulating the morphology of the plasmonic metals. Therefore, the above‐mentioned three photothermal effect mechanisms are suitable for different photothermal materials with specific natures.^[20]^ Photothermal catalysis, which refers to reactions containing the essential factors of light, heat, and catalytic conversion, can be divided into three types according to the different reaction pathways: light‐driven thermal catalysis, thermal‐assisted photocatalysis, and photothermal synergistic catalysis.

#### 2.2.1 Light‐Driven Thermal Catalysis

Light‐driven thermal catalysis is essentially a traditional thermal catalytic reaction process, in which the thermal energy comes from the solar energy rather than the consumption of fuels. In this photocatalytic process, light only plays the role of heat‐energy supplier to trigger the reactions. The Fischer–Tropsch synthesis (FTS) is a typical thermal catalytic reaction, which can directly convert syngas (CO and H_2_) into olefins.^[21]^ During the FTS catalytic process, significant energy input is necessary, which usually comes from the consumption of fossil fuels or from electricity. In recent years, as a result of the energy crisis, exploring green, low‐energy input technologies for promoting the FTS catalytic process has become an increasing trend. Inspired by the photothermal effect of metallic nanoparticles (Fe, Co, Ni etc.) in traditional FTS catalysts, which have the capacity to convert light into thermal energy, a strategy for promoting the FTS by using light as the external energy source, called light‐driven FTS, has recently been proposed.

**Figure 2:**
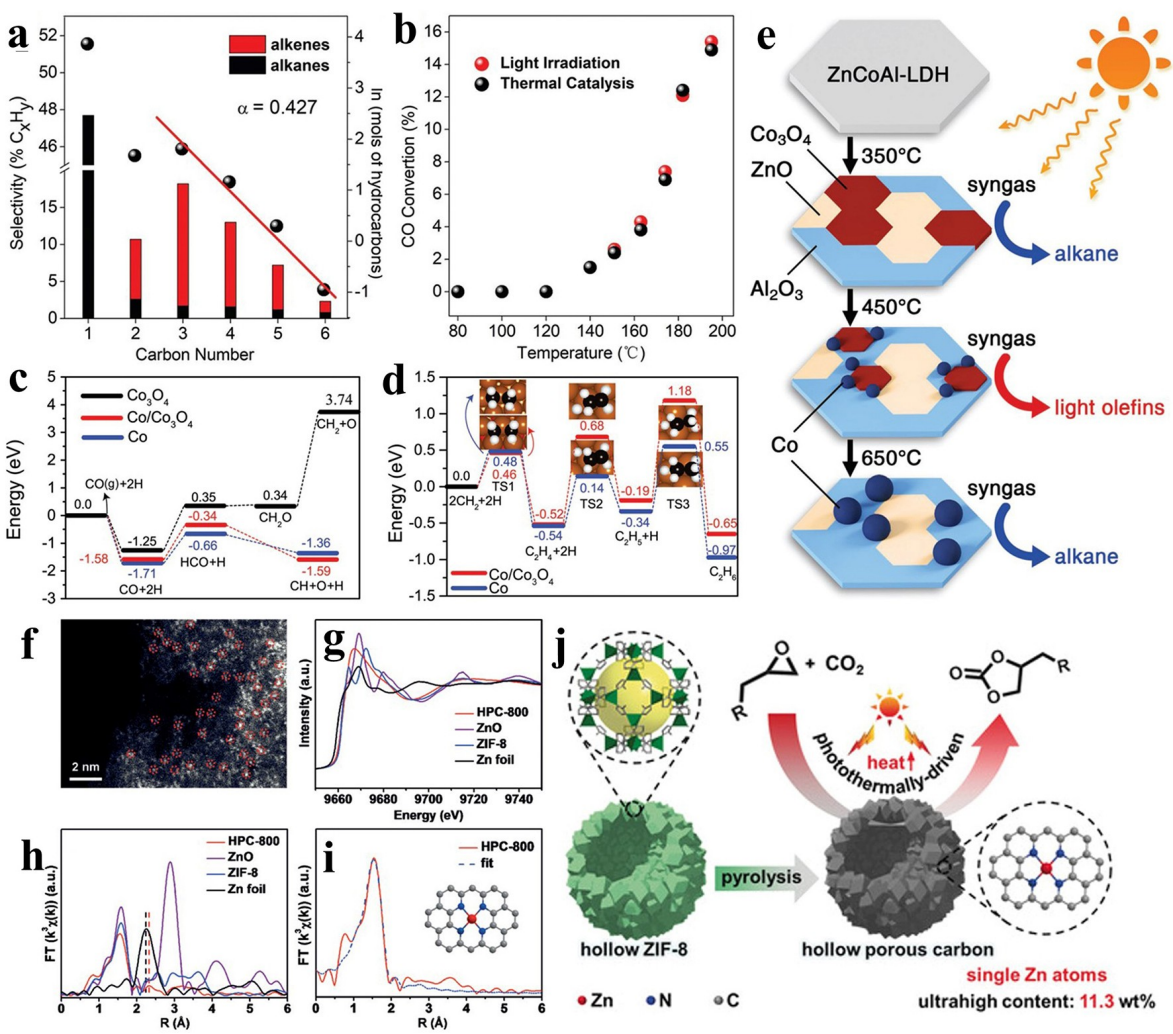
a) Distribution of hydrocarbon products obtained over Co‐450 under irradiation with UV/Vis light. b) Comparison of CO conversion for Co‐450 under photothermal heating (UV/Vis irradiation) and direct thermal heating (no UV/Vis irradiation). c) Potential energy profile of CO dissociation on Co_3_O_4_(220), Co(111)/Co_3_O_4_(220), and Co(111). d) CH_2_ coupling and C_2_H_4_ hydrogenation on Co(111)/Co_3_O_4_(220) and Co(111). e) Fabrication of Co‐*x* catalysts by the reduction of ZnCoAl‐LDH nanosheets with H_2_ at 300–700 °C, and the selectivity of their products for CO hydrogenation under irradiation with UV/Vis light. Reproduced with permission.^[22]^ Copyright 2018, Wiley‐VCH. f) Aberration‐corrected HAADF‐STEM image of HPC‐800 (single Zn atoms are highlighted with red circles). g) Zn K‐edge XANES and h) k3‐weighted Fourier transform of the EXAFS spectra. The dashed lines highlight the peak difference of the Zn foil and HPC‐800. i) EXAFS R‐space fitting curve of HPC‐800 (inset: schematic model of HPC‐800: Zn red, N blue, and C gray). j) Schematic illustration showing the fabrication and catalytic process of the HPC. Reproduced with permission.^[24]^ Copyright 2019, Wiley‐VCH.

One typical example was reported by the Zhang group. By using ZnCoAl‐LDH as the precursor, they successfully constructed different kinds of Co‐based photothermal catalysts through regulating the H_2_ reduction temperatures (Figure 2a–e). The catalyst prepared at 450 °C (Co‐450), composed of a Co/Co_3_O_4_ composite growing on a zinc oxide/alumina substrate, displayed the highest Tropsch to olefins (FTO) performance, with an olefin (C_2–4_^=^) selectivity of 36.0 % under irradiation with UV light. Benefitting from the presence of Co nanoparticles, which can harvest light over a wide wavelength, the light was converted into heat to promote the FTS. Moreover, from the combination of structural analysis and theoretical calculations, the commendable selectivity of Co‐450 toward olefins can be explained by the heterostructure formed between Co and Co_3_O_4_ nanoparticles weakening the hydrogenation ability of the Co nanoparticles, thus enhancing the selectivity.^[22]^ Inspired by above work, the Zhang group also successfully constructed MnO‐supported nickel photothermal catalysts by a H_2_ reduction process. Under UV/Vis irradiation, the prepared photothermal catalysts exhibited remarkable activity and selectivity for CO hydrogenation to produce olefins. In the composite, the Ni acted as the light harvester to input thermal energy, and the MnO acted as the electron donor to accumulate electrons on the Ni nanoparticles, thus restricting the degree of surface hydrogenation and increasing the selectivity.^[23]^

In addition to the FTS reaction, the traditional thermal catalytic cycloaddition of CO_2_ can also be induced by the thermal energy provided by the light. By utilizing the typical MOF ZIF‐8 as the precursor, Jiang and co‐workers successfully fabricated a series of nitrogen‐doped hollow porous carbons (HPCs) decorated with Zn single atoms. The advantages of the prepared HPCs can be summed up in the following points: 1) The carbons possess a photothermal effect, and can effectively absorb light to produce heat, and their porous structure can induce the multiple reflection of incident light, thus further improving the photothermal performance; 2) the rich N chelation sites on HPCs are beneficial for anchoring the Zn single atoms; 3) the porous structure of HPCs can contribute to enriching the CO_2_ molecules and facilitating the transmission of substrates/products. Consequently, HPCs with the proper calcination temperatures can effectively catalyze the cycloaddition of CO_2_ under irradiation with light (Figure 2f–j).^[24]^ In addition to carbon species, other materials, such as MOFs and plasma metals, can also be used as light harvesters to convert light into heat and promote the CO_2_ cycloaddition reaction. Among them, MOFs are a kind of emerging photothermal material which has attracted tremendous interest in recent years. For example, Peng and co‐workers found that the Zr‐ferrocene (Zr−Fc) MOF displayed an excellent photothermal effect, as a result of the incorporation of ferrocene‐based ligands. Therefore, by using the Zr−Fc as the photothermal conversion material and Keggin‐type polyoxometalates (POMs) with abundant Lewis acid sites as the CO_2_ cycloaddition catalyst, PMo_12_@Zr−Fc MOF nanosheet composites were successfully constructed that showed a high performance for the solar‐driven CO_2_ cycloaddition. Under irradiation with light at an intensity of 0.4 W cm^−2^, the PMo_12_@Zr−Fc composite can rapidly raise the reaction temperature up to 80 °C, and effectively convert the CO_2_ and styrene oxide into styrene carbonate with an excellent conversion efficiency of 88.05 %.^[3]^

#### 2.2.2 Thermal‐Assisted Photocatalysis

The essence of thermal‐assisted photocatalysis is a photocatalytic process consisting of the excitation of electrons and generation of hot carriers. In this catalytic system, thermal energy generated from the catalysts plays the role of accelerating mass transfer and the adsorption/desorption rate, lowering the activation energy of the reaction, and promoting the migration/separation of charge carriers, all of which contribute to boosting the photocatalytic reaction. The generated thermal energy only constitutes a small fraction of the absorbed light energy, with most of the energy used for exciting electrons so that they migrate to the conduction band.[^19^, ^25^]

**Figure 3:**
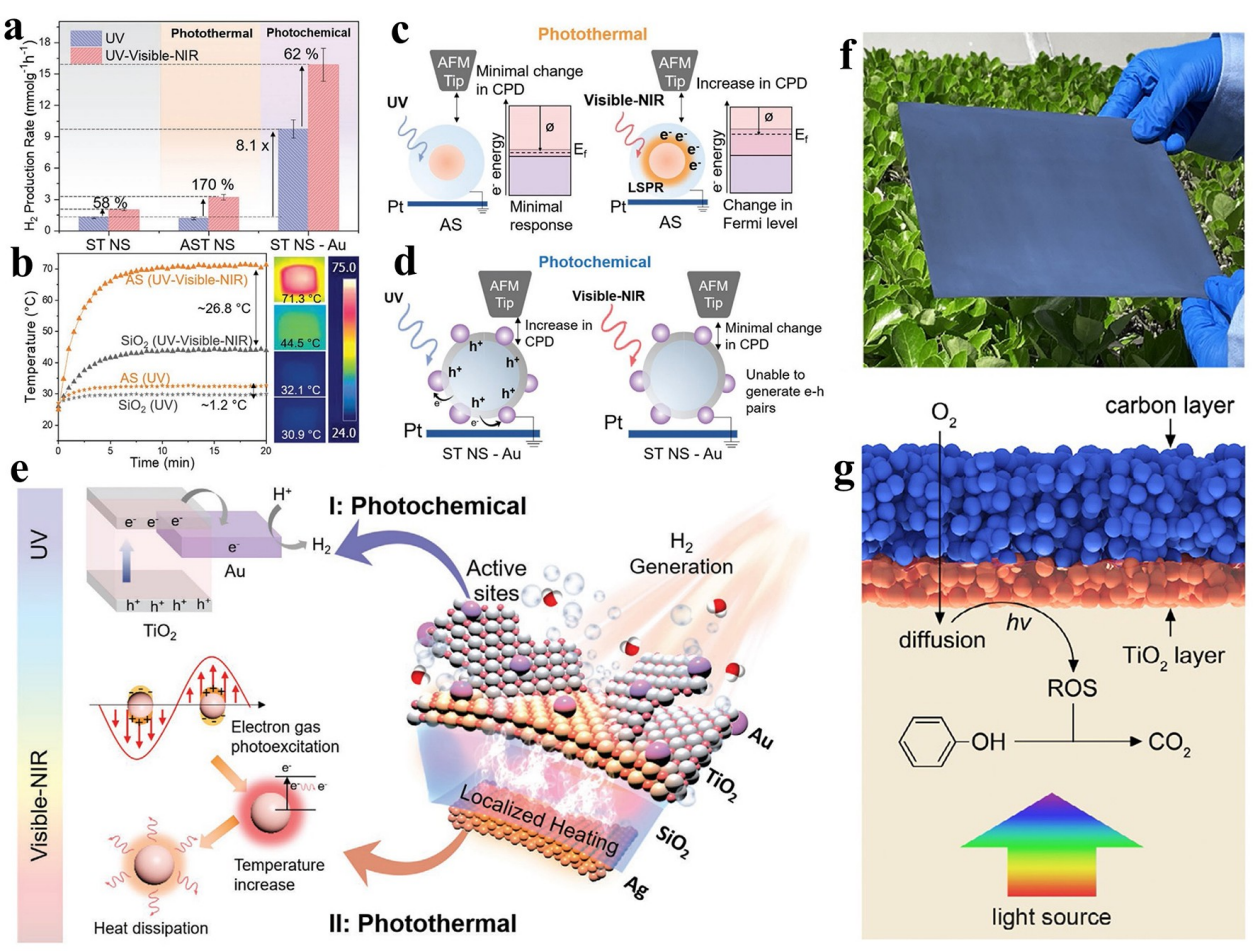
a) H_2_ generation rates of ST NS, AST NS, and ST NS−Au under irradiation with UV light and full spectrum light. b) Temperature profile of SiO_2_ and AS under irradiation with UV light and full spectrum light. c) Schematic diagram showing the effect of irradiation with UV and Vis/NIR light on the surface potential of AS. d) Schematic diagram showing the effect of irradiation with UV and Vis/NIR light on the surface potential of ST NS−Au. e) Schematic diagram illustrating the mechanism of enhanced photocatalytic H_2_ generation as a result of photothermal and photochemical effects. Reproduced with permission.^[26]^ Copyright 2021, Wiley‐VCH. f) Photograph of a TiO_2_/C bilayer paper, size: 25 cm×25 cm. g) Schematic illustration of the photothermal‐assisted triphasic photocatalytic oxidation of phenol. Reproduced with permission.^[27]^ Copyright 2021, Wiley‐VCH.

To achieve thermal‐assisted photocatalysis, the photothermal materials, which can convert light into thermal energy, are usually integrated with semiconductor photocatalysts. For example, Ho and co‐workers successfully designed Ag@SiO_2_@TiO_2_−Au (ASTNS−Au) photothermal catalysts with core–shell structures (Figure 3a–f). In this hybrid photothermal catalyst, the Ag@SiO_2_ serves as the core, with the Ag nanoparticles absorbing photons of low energy (Vis/NIR) and releasing the heat through an electron–electron scattering process arising from the LSPR effect, and the SiO_2_ layer increasing the refraction of light, thus increasing the light‐heating performance of the Ag core. The shell was composed of TiO_2_ and Au nanoparticles, whereby the TiO_2_ can be excited by the UV light to generate electron–hole pairs to induce the redox reactions, and the Au nanoparticles can act as co‐catalysts to prevent the recombination of electron–hole pairs. Benefitting from the well‐designed core–shell structure, the ASTNS−Au can make full use of light over a wide wavelength and can exhibit exceptionally high photocatalytic activity for water splitting, with the H_2_ evolution rate reaching 30.2 mmol g^−1^ h^−1^.^[26]^

Carbon‐based materials are another kind of widely investigated photothermal materials. Selecting TiO_2_ as the photocatalyst and carbon as the photothermic material, the Zhang group recently reported a scalable bilayer paper with Janus wettability, which can effectively facilitate the generation of photochemical reactive oxygen species (ROS) under full spectrum irradiation (Figure 3e–g). During the photocatalytic process, the bilayer paper floats on the surface of the reaction solution, thus forming a gas–liquid–solid triphasic photocatalytic system. In comparison with the diphase system with dispersed nanoparticles, such a triphasic system displays two main advantages for the photochemical generation of ROS. First, the triphasic system uses O_2_ from the gas phase as the reactant, rather than dissolved O_2_ from the liquid phase, thus facilitating the diffusion of oxygen. Second, in the triphasic system, the heat generated by the carbon nanoparticles can be directly transferred to the surface of the TiO_2_, rather than delivering heat through the aqueous medium, which greatly reduces the dissipation of the heat energy. Therefore, the bilayer paper can rapidly increase the temperature to 55 °C in the local area of the carbon layer within one minute under full spectrum irradiation, which not only accelerates the charge transfer, but also improves the free radical reaction kinetics. Consequently, the carefully designed three‐phase thermal‐assisted photocatalytic system based on a TiO_2_/C bilayer paper displayed excellent degradation performance for the oxidation of phenol.^[27]^ Moreover, such bilayer paper can also be used in a triphasic flow reactor and shows great potential in large‐scale photocatalytic applications.

Based on the above considerations, the authors also designed a triphasic photocatalytic CO_2_ reduction system by placing the Ag‐TiO_2_ layers at a gas–water interface. It is worth mentioning that such triphasic systems were also applicable in dilute CO_2_ environments, which further prove their practicality.^[28]^ Apart from the oxidation of phenol and reduction of CO_2_, the triphasic catalytic system can also be applied for solar‐driven oxygen reduction to produce hydrogen peroxide. In previous studies, traditional semiconductor materials, such as CdS, TiO_2_, as well as g‐C_3_N_4_, were often selected as the photocatalysts to produce hydrogen peroxide. However, their photocatalytic activity for the reduction of O_2_ is still low, and the addition of alcohols as sacrificial agents is still indispensable, which makes the separation of products more difficult. In this regard, Yu and co‐workers successfully prepared a covalent organic framework (COF) based photocatalyst (TPB‐DMTP‐COF) that can effectively convert O_2_ into H_2_O_2_ under irradiation with visible light over a wide range of pH values without the addition of sacrificial agents. Theoretical calculations also confirmed that the triphenylbenzene group should be the active sites because of their high charge density. More importantly, as a consequence of its hydrophobic properties, the TPB‐DMTP‐COF can be coated onto a porous carbon fiber paper, thus forming a gas–liquid–solid triphasic photocatalytic system. The triphasic photocatalytic system can effectively improve the O_2_ concentration and transfer rate, and improve the charge transfer because of the good electrical conductivity of the porous carbon fiber. Moreover, the porous carbon can also convert light into thermal energy to further increase the mass transfer and separation/migration of the electron–hole pairs. Thus, the triphasic photocatalytic system effectively promoted the reaction of hydrogen peroxide production, with a record rate for the generation of H_2_O_2_.

#### 2.2.3 Photothermal Synergistic Catalysis

Similar to thermal‐assisted photocatalysis, photothermal synergistic catalysis also consists of the excitation of electrons and generation of hot carriers. However, photothermal synergistic catalysis is an integrated photocatalytic process, which is composed of both thermochemical and photochemical pathways. Moreover, the generated heat will constitute a large portion of the absorbed light energy, thus influencing the reaction mechanism through exciting the vibrations or phonons.^[29]^ In contrast to the photocatalytic process of CO_2_ reduction, the photothermal catalytic reaction of CO_2_ and H_2_ to produce CH_4_ (Sabatier reaction) is more efficient and can sometimes even be achieved under relatively mild conditions.

**Figure 4:**
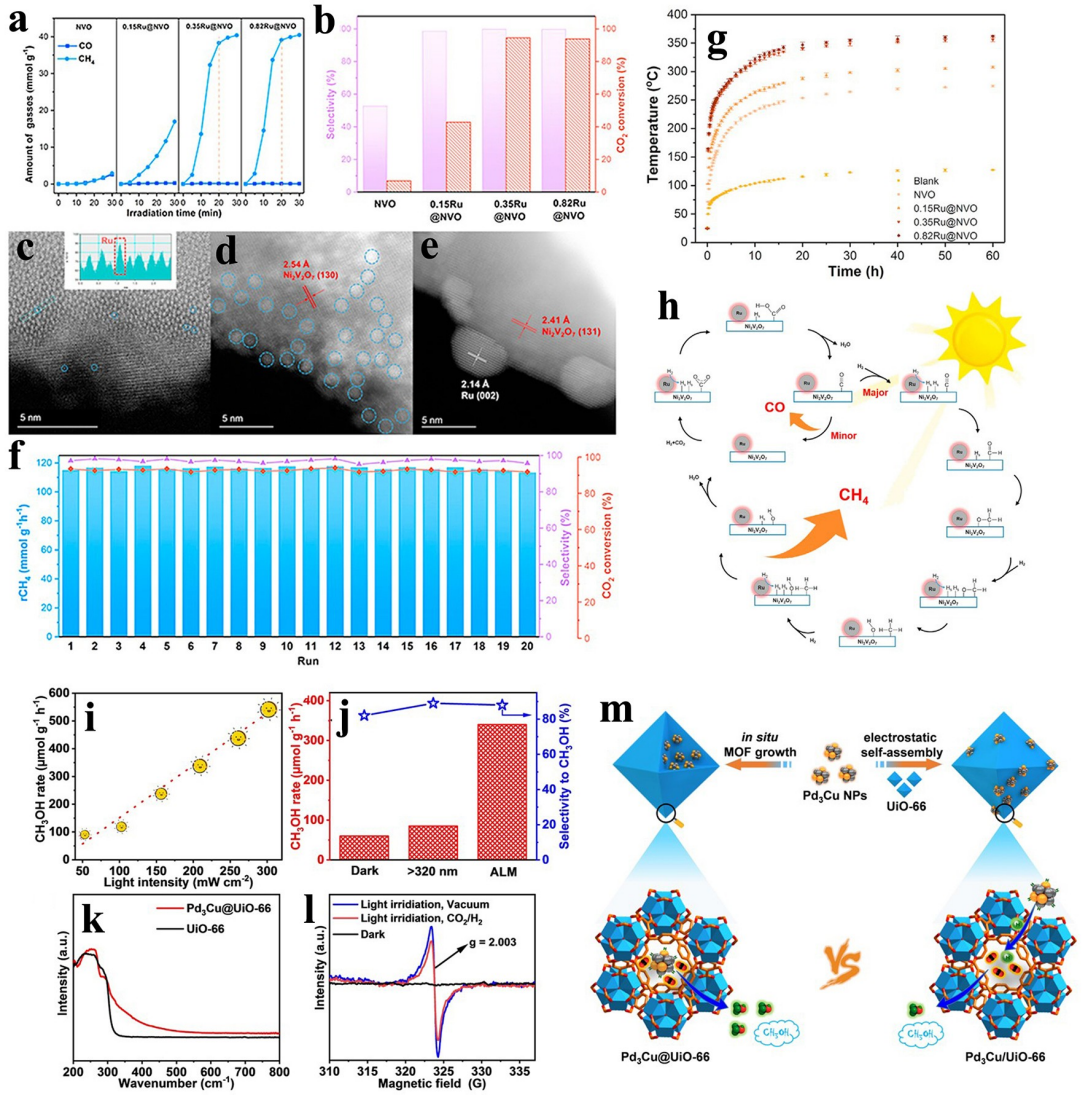
a) CO_2_ conversion as a function of irradiation time under a 300 Xe lamp with an optical density of 2.0 W cm^−2^. b) CH_4_ selectivity and CO_2_ conversion. c)–e) HAADF‐STEM images of c) 0.15Ru@NVO, d) 0.35Ru@NVO, and e) 0.82Ru@NVO. f) Stability testing for the catalytic generation of CH_4_ over 0.35Ru@NVO. g) Variation in the temperatures of different NVO‐based catalysts under a full‐Arc 300 W Xe lamp with an optical density of 2.0 W cm^−2^. The error bars represent the standard deviation of five independent measurements on the same catalyst. h) Proposed catalytic reaction mechanism for the hydrogenation of CO_2_ over the 0.35Ru@NVO catalyst. Reproduced with permission.^[30]^ Copyright 2021, Wiley‐VCH. i) Dependence of the methanol production rate on the light intensity in the presence of the Pd_3_Cu@UiO‐66 catalyst. j) Methanol formation rates over Pd_3_Cu@UiO‐66 in the dark or under irradiation at >320 nm or with full‐spectrum light. k) UV/Vis spectra of UiO‐66 and the Pd_3_Cu@UiO‐66 catalyst. l) ESR spectra of Pd_3_Cu@UiO‐66 under different conditions. m) Schematic illustration showing the light‐assisted hydrogenation of CO_2_ to CH_3_OH over Pd_3_Cu@UiO‐66 and Pd_3_Cu/UiO‐66, whereby H_2_ and CO_2_ molecules are activated on Pd_3_Cu NPs and defective Zr‐oxo clusters, respectively, in proximity in Pd_3_Cu@UiO‐66, which leads to its enhanced activity. Reproduced with permission.^[31]^ Copyright 2021, Wiley‐VCH.

Recently, Li and co‐workers reported a ruthenium‐supported nickel metavanadate catalyst (0.35 %Ru@Ni_2_V_2_O_7_), which coupled photochemical/photothermal reactions for promoting light‐driven Sabatier transformations.^[30]^ The Ru@Ni_2_V_2_O_7_ displayed a high light harvesting performance in the UV, Vis, and NIR regions. The Ru clusters played the role of nanoheaters, which increased the local temperature to activate H_2_ and dissociate H_2_O molecules, with the released H atoms reacting with CO_2_ adsorbed on the oxygen vacancies of Ni_2_V_2_O_7_, thereby realizing the photothermal synergistic catalytic methanation of CO_2_. Decorating the Ni_2_V_2_O_7_ with Ru clusters results in the energy barrier for CO_2_ activation being significantly reduced through stabilization of the COOH* intermediate, while the formation energy of CHO* can be modified at the same time, thus effectively regulating the reaction pathways and enhancing the selectivity of CH_4_ production. Moreover, Ni_2_V_2_O_7_ also functioned as a semiconductor photocatalyst, with the ability to convert CO_2_ into CH_4_ through a photochemical pathway. Consequently, under the synergistic effect of photocatalysis and thermocatalysis, the Ru@Ni_2_V_2_O_7_ exhibited a high efficiency and selectivity for CO_2_ conversion, with a record solar‐driven CO_2_ methanation rate of 114.9 mmol g_cat_^−1^ h^−1^ (Figure 4a–h).

As a consequence of their porous frameworks, tailored structures, and high BET surface areas, metal‐organic frameworks have been considered as good candidates for preparing multifunctional catalysts. Thus, the Jiang group took advantage of the high stability and porous structure to successfully encapsulate tiny Pd_3_Cu nanoparticles into the classical UiO‐66 MOF material. Although Pd_3_Cu is the main photothermal catalyst, which can convert light energy into heat and trigger the CO_2_ hydrogenation reaction in this composite, the UiO‐66 also plays a crucial role in promoting CO_2_ activation and HCOO* formation through delivering the photogenerated electrons on the MOF to the antibonding orbitals of CO_2_*. Interestingly, they also found that the location of the Pd_3_Cu (in or on the MOF materials) can greatly influence their activity for converting CO_2_ into CH_4_. It is worth mentioning that this is the first example of using MOFs for the hydrogenation of CO_2_ (Figure 4i–m).^[31]^

In addition to the above examples, the photochemical and thermochemical pathways in photothermal synergistic catalysis can sometimes also occur sequentially. A typical example was reported by Cen and co‐workers.^[29f]^ By using a sol–gel process, they constructed a series of M‐doped TiO_2_ films (M=Zn, Ni, and Cu) to investigate the CO_2_ conversion. Among them, Cu‐doped TiO_2_ can most effectively promote the CO_2_ conversion, with the CO production rate reaching up to 10.80 μmol g^−1^, approximately 6.39 times greater than that of clean TiO_2_. The reaction mechanism was determined by a series of experimental and theoretical calculations that showed the photoexcited electron–hole pairs of the photoreaction played the decisive role in the generation of oxygen deficiency, and that the photoinduced oxygen vacancies played an important role in converting CO_2_ into CO in a thermal reaction.

Consequently, these three types of photothermal catalytic reactions show differences and similarities. The biggest difference between light‐driven thermal catalysis and thermal‐assisted photocatalysis is the reaction pathways, with the light‐driven thermal catalysis being essentially a thermochemical pathway, while the thermal‐assisted photocatalysis follows a photochemical pathway. A comparison of photothermal synergistic catalysis and thermal‐assisted photocatalysis shows that both of them consist of electron excitation and thermal generation, but the thermal energy in thermal‐assisted photocatalysis only constitutes a small fraction of the absorbed light energy, with most energy being used for exciting electrons. In contrast, thermal generation constitutes the majority of the energy in photothermal synergistic catalysis. The similarity between all three types of photothermal catalytic reactions is that they all harvest light to promote the reactions.

### 2.3 Solar Cell Powered Electrocatalysis

Photocatalysis, photoelectrochemical catalysis, and solar cell powered electrocatalysis have been considered as efficient technologies for converting light energy. All of them utilize light energy to induce many catalytic reactions, and finally convert the light into chemical energy under mild conditions, but some differences exist between them. In photocatalysis and photoelectrochemical catalysis, the semiconductors are used as the major active components, but a bias potential should be applied in photoelectrochemical systems to restrict the recombination of the electron–hole pairs and, thus, promote the photocatalytic redox process. Moreover, the reaction conditions for these two systems also differ, whereby photocatalysis is performed in a “particle‐based system” composed of semiconductor powders in a reaction solution or suspension, whereas photoelectrochemical catalysis is performed on photoactive thin film(s) that function as the photoelectrode in the presence of an electrolyte.

**Figure 5:**
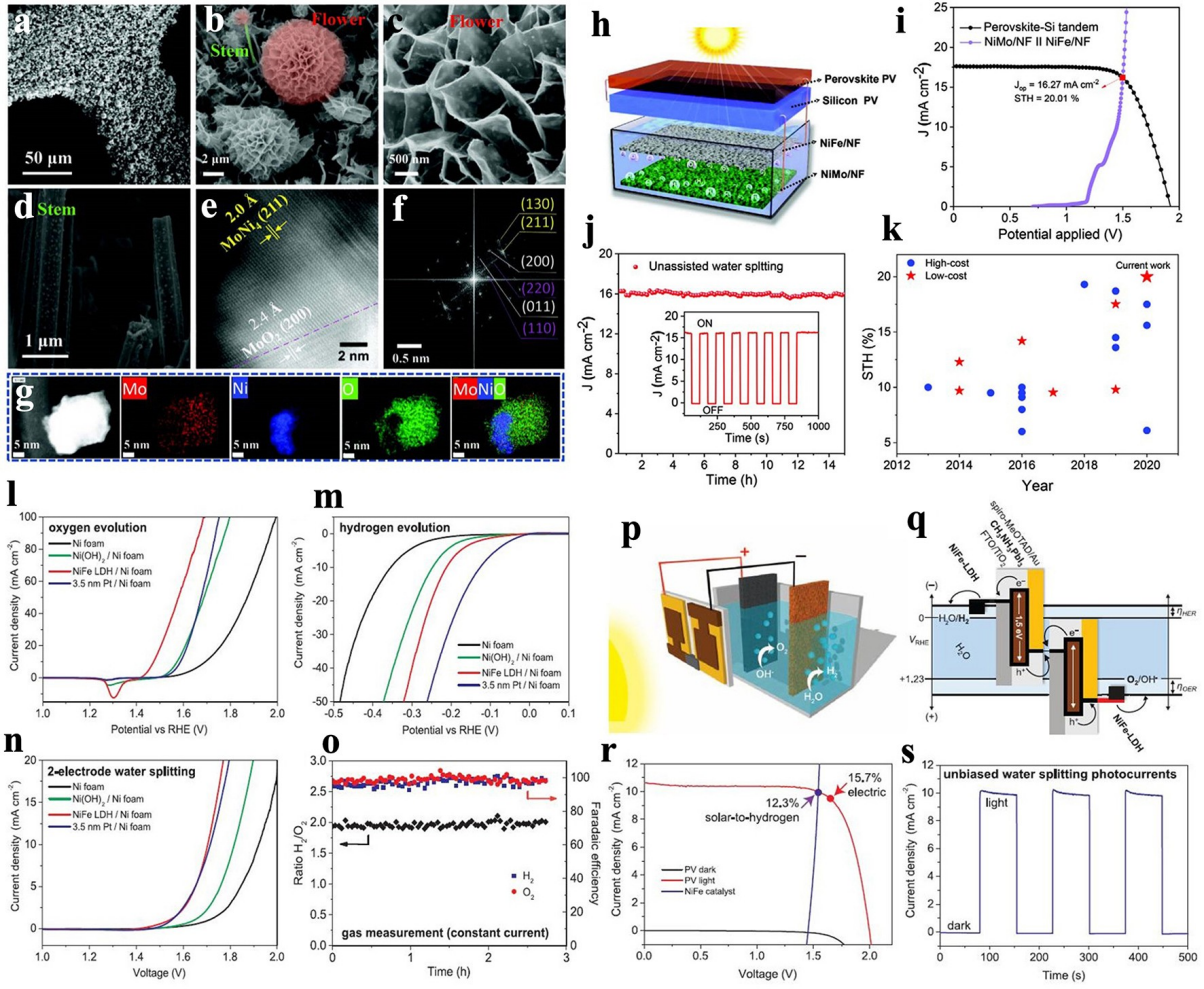
Morphology and chemical composition of NiMo/NF. a)–d) SEM images, e) HAADF‐STEM image, f) FFT image, where crystal facets of MoNi_4_, MoO_2_, and NiMoO_4_ are shown in yellow, white, and purple, respectively, and g) EDX mapping of a nanoparticle on the stem‐like structure. h) Schematic illustration of the direct solar water splitting system consisting of a perovskite‐Si tandem cell integrated with NiFe/NF||NiMo/NF electrodes. i) Overlay of the *J*–*V* curve of a perovskite‐Si tandem cell with the LSV curve of the NiMo and NiFe electrodes in a two‐electrode configuration. j) Unassisted water splitting current generated by the integrated system. k) Comparison of the STH efficiency achieved in this work with reported values for other solar‐driven water‐splitting systems. Reproduced with permission.^[33]^ Copyright 2014, The American Association for the Advancement of Science. l)–o) Electrochemical performance of different catalyst electrodes by linear sweep voltammetry in 1 m NaOH aqueous electrolyte, and gas chromatographic measurement of gases evolved from NiFe LDH electrodes. p) Schematic diagram of the water‐splitting device. q) A generalized schematic illustration of the energy of the perovskite tandem cell for water splitting. r) *J*–*V* curves of the perovskite tandem cell under dark and simulated AM 1.5G 100 mW cm^−2^ illumination, and the NiFe/Ni foam electrodes in a two‐electrode configuration. s) Current density/time curve of the integrated water‐splitting device without external bias. Reproduced with permission.^[34]^ Copyright 2021, Wiley‐VCH.

Solar cell powered electrocatalysis is another solar conversion process. It consists of a combination of solar cells and electrocatalytic reactions, and is realized through a two‐step energy conversion pathway. Light energy is firstly efficiently transformed into electrical energy and then the generated electrical energy is used to drive the electrocatalytic reactions. Electrocatalysts with excellent conductive properties are used as the major active components in solar cell powered electrocatalysis system, whereas semiconductors are employed in photocatalytic and photoelectrochemical catalytic systems. Although solar cell powered electrocatalysis is not a one‐step direct utilization of solar energy, its reaction efficiency is still higher than the other two catalytic reactions. Moreover, it can also help to solve the issue of electric energy being hard to store. The solar cell powered electrocatalytic reaction is a continuous energy‐conversion process, and the overall efficiency of the reaction system depends not only on the high‐performance solar cells and electrode thin films, but also on their degree of matching and stability. Along with the rapid advancements in the photovoltaic industry, taking Si as a typical example, the power conversion efficiency (PCE) of solar cells has been increased from 15 % to 26 %. Thus, in recent years, solar cell powered electrocatalysis has gradually attracted tremendous interest from academia and industry, and remarkable achievements have also been achieved.[^5^, ^32^] As far as we know, solar cell powered electrocatalysis is mainly studied for its ability to effect water splitting and CO_2_ reduction. Just as its name implies, solar cell powered electrocatalytic water splitting is achieved by coupling solar cells and water electrolysis devices. For example, by integrating perovskite photovoltaics and earth‐abundant electrocatalysts, Luo et al. successfully developed a low‐cost, high‐performance solar cell powered water splitting system. In this system, the solar cell was based on CH_3_NH_3_PbI_3_ perovskite films, constructed by a two‐step coating strategy, and possess a state‐of‐the‐art PCE of 17.3 %, and the electrocatalysts were composed of bifunctional NiFe‐layered double hydroxides (LDHs)/Ni foam (NiFe LDH/Ni foam), constructed by a simple hydrothermal method, with both remarkable H_2_ and O_2_ evolution reaction (HER and OER) activities in alkaline electrolytes. Benefitting from the remarkable properties of the photovoltaics, in which the open‐circuit voltage, photocurrent density, and the fill factor are 1.06 V, 21.3 mA cm^−2^, and 0.76, respectively, only applying two cells was enough to drive the electrocatalytic splitting of water. Although, the HER activity of the NiFe LDH/Ni foam was lower than that of the Pt/Ni foam electrode, it displayed a higher activity for the OER, in which the required overpotential of the NiFe LDH/Ni foam was 100 mV lower than that of the Pt/Ni foam. Despite the lower HER activity in strong base, the electrocatalytic activity of the NiFe LDH/Ni foam was sufficiently good. Moreover, the cost of the NiFe LDH/Ni foam was much lower than the Pt/Ni foam. Therefore, it was suitable for application in a solar cell powered electrocatalytic system. Consequently, the coupling of perovskite photovoltaics and earth‐abundant electrocatalysts led to a solar water splitting system that displayed an outstanding solar‐to‐hydrogen (STH) efficiency (12.3 %) and a photocurrent density as high as 10 mA cm^−2^ (Figure 5a–k).^[33]^

To achieve a higher STH efficiency, various solar cell powered electrocatalytic systems were developed in the subsequent years. Although many solar water‐splitting systems display a higher STH efficiency, with some of them even reaching up to approximately 30 %, most of the excellent results are obtained with high‐cost semiconductors and noble metals. In this regard, the Zhao group successfully developed a low‐cost solar cell powered electrocatalytic system by integrating perovskite/Si tandem semiconductors with Ni‐based electrocatalysts. The electrocatalytic system was composed of NiMo alloy as the HER catalyst and NiFe alloy as the OER catalyst. The unique flower‐stem morphology of the NiMo catalysts, which was beneficial for enhancing the interactions between the catalysts and electrolyte and improving the reaction kinetics and preventing the difficult desorption of gas products, led to the kinetics of the water reduction reaction being significantly improved. The NiFe alloy was also demonstrated to be an efficient catalyst for the OER with a low overpotential (255 mV at 10 mA cm^−2^). Photovoltaics were developed to provide the electrical energy and promote the overall water splitting. The perovskite film was passivated by the *n*‐dodecylammonium bromide, a large organic cation with a long alkyl chain, which can make contributions to reducing the surface defects and regulating the band structure, thus enhancing the power conversion efficiency. Therefore, through combining the efficient solar cells with NiMo HER and NiFe OER electrocatalysts, the catalytic systems effectively promoted water splitting with a record STH of 20 %. Moreover, the levelized cost of hydrogen was reduced to about $3 kg^−1^ (Figure 5l–s).^[34]^

**Figure 6:**
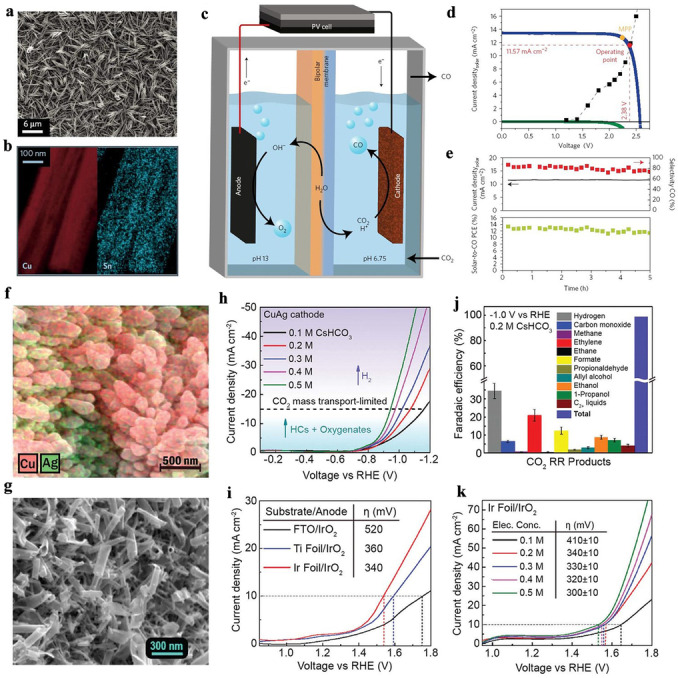
a) Scanning electron micrograph of as‐prepared CuO nanowires. b) X‐ray diffraction pattern of SnO_2_‐modified and unmodified samples. From the annotated reflections, the presence of CuO could be observed but no reflections corresponding to SnO_2_ were detected. c) Schematic illustration of the solar‐driven device for CO_2_ reduction. d) Photovoltaic and electrocatalytic *J*–*V* behaviors. e) Selectivity toward CO, solar current density, and solar‐to‐CO efficiency as a function of photoelectrolysis time. Reproduced with permission.^[35]^ Copyright 2017, Springer Nature. f) EDX elemental mapping of the CuAg nanocoral cathode. g) SEM surface view of IrO_2_ nanotubes on iridium foil. h) *J*–*V* curves of the CuAg nanocoral cathode under various electrolyte conditions. i) *J*–*V* curves of the IrO_2_ nanotube anode on FTO, Ti foil, and Ir foil substrates in 0.2 m CsHCO_3_ electrolyte. j) CO_2_ RR product distribution at −1 V vs. RHE (ca. 10 mA cm^−2^) in 0.2 m CsHCO_3_ electrolyte. Error bars are standard deviations based on replicate experiments. k) *J*–*V* curve of the Ir foil/IrO_2_ anode under various electrolyte conditions. Electrochemical measurements were performed under constant CO_2_ bubbling (the conditions used in the electrolysis cell). Reproduced with permission.^[36]^ Copyright 2017, Royal Society of Chemistry.

In addition to water splitting, solar cell powered electrocatalysis can also be applied to CO_2_ reduction. For example, through atomic layer deposition, Schreier and co‐workers successfully fabricated CuO nanowires decorated with a small amount of SnO_2_, and these were applied as bifunctional catalysts, as both the cathode and anode, for achieving overall CO_2_ reduction and to produce CO and O_2_, respectively. The presence of surface‐modified SnO_2_ was indicated by a series of spectral tests. The SnO_2_ helps weaken the interactions between the catalysts and CO products and H*, thus improving the electrocatalytic activity and selectivity for CO_2_ reduction. With the high‐performance catalysts in hand, a triple‐junction GaInP/GaInAs/Ge photovoltaic was selected as the power source to induce the reaction, and a bipolar membrane was used to separate the electrodes, thus enabling the half‐redox reactions to occur under optimized electrolyte conditions. Consequently, a solar‐driven CO_2_ reduction device was successfully assembled by combining the bipolar membrane separated electrodes and high‐performance solar cells. Driven by illumination with simulated light, the solar to CO efficiency of the device can reach up to 13.4 % with 81 % faradaic efficiency under an operating voltage of 2.38 V.^[35]^ In addition to the above examples, some other devices have also been designed that can achieve solar powered electrocatalytic CO_2_ reduction, but most of them depend on noble metal electrocatalysts (Figure 6a–e). Therefore, this study provided a new insight for designing highly efficient solar driven CO_2_ reduction devices with a low cost.

Although some devices have been reported that can achieve solar‐driven CO_2_ reduction, their products are mainly CO and formate, which requires further fabrication procedures before use as a fuel. Therefore, constructing more efficient solar‐driven devices to convert CO_2_ into highly reduced products, including methane, ethylene, and oxygenates, through multielectron transfer is urgently required. To achieve this goal, the Joel group developed an efficient sandwich‐type electrochemical cell coupled with a tandem photovoltaics system (Figure 6f–k). In the electrochemical cell, the CuAg bimetallic cathode was designed to trigger the CO_2_ reduction reaction (CO_2_RR) because of its wide operational window and high selectivity. IrO_2_ nanotubes were applied at the anode to promote water oxidation, and CsHCO_3_ with its excellent selectivity for C_2_ products was selected as the electrolyte. Consequently, after integrating such an electrochemical cell with two series‐connected silicon solar cells and a maximum power point (MPP) tracker, an efficient solar driven electrochemical system was obtained. It was worth mentioning that the high efficiency even exceeded that of natural photosynthesis.^[36]^ Solar cell powered electrocatalysis is a continuous energy‐conversion process, and the overall efficiency depends not only on the high‐performance of the solar cells and electrode materials, but also on their degree of matching and their permanent stability. When constructing a reaction system, various parameters need to be optimized to match the battery and electrodes, thus reducing the energy consumption, and improve the performance and stability of the solar cell powered electrocatalytic systems.

### 2.4 Pyroelectric Catalysis

Temperature vibration derived from the movement of the sun is a common phenomenon in our daily life, and it can also be used as a type of clean and abundant energy source. Therefore, there is an urgent requirement to explore ideal materials that can efficiently convert it into chemical fuels. Pyroelectric materials are a subclass of piezoelectric materials with an asymmetric structure and exhibiting spontaneous polarization that usually crystallize in the 1, 2*, m, mm*2, 4, 4*mm,* 3, 3*m,* 6, and 6*mm* point groups. Temperature vibration will cause a position offset of the central atom, thus changing the polarity of the pyroelectric materials. At the same time, a pyroelectric current will also be generated, which can be calculated by Equation 4:

(4) I=dQ/dt=PcA(dT/dt)

In this equation, d*T*/d*t* stands for the temperature vibration rate, Pc represents the pyroelectric coefficient under short circuit conditions, *A* stands for the surface area, and *I* stands for the pyroelectric current of the pyroelectric materials. According to the equation, we know that the pyroelectric current intensity relies on the pyroelectric coefficient of the materials, their surface area, as well as variation of the ambient temperature.^[4]^ The temperature has a great impact on the pyroelectric materials, which will be able to display pyroelectric properties only when the temperature is below the Curie temperature (*T*_c_). When the temperature fluctuates (d*T*/d*t*>0 or d*T*/d*t*<0), the polarization of the pyroelectric material will be changed, thus causing a break in the electrical balance and bound polarization charges, thereby generating pyroelectric free charges on their surfaces with a built‐in electric field. As a consequence of the above features, pyroelectric materials can not only accelerate the migration of the charge carriers and promote the photocatalytic reaction, but can also generate the pyroelectric positive and negative charges to trigger the reaction, similar to electrocatalysis. Therefore, the pyroelectric catalytic reaction will be promoted by the conversion of energy from temperature vibration energy to electrical energy and then to chemical energy. Currently, the thermal energy input is derived in two ways: either by artificially controlling the temperature through a circuit controller or induced by the changes in the day and night temperatures. The latter method is more energy saving and is not restricted by the experimental conditions. However, related studies have rarely been reported. One fascinating example was reported by Zhang et al. In their study, the influence of the electrolyte concentration and the temperature vibration frequency on the pyroelectric water splitting performance was systematically studied. Benefitting from the pyroelectric effect in PbTiO_3_, positive and negative charges were induced by temperature vibration to generate the electrical energy for the electrocatalytic splitting of water. It was demonstrated that a voltage of 2.34 V would be produced when the working electrodes were connected with a PbTiO_3_ pyroelectric harvester in 0.5 m KOH electrolyte, which was strong enough for the electrocatalytic production of H_2_. Moreover, by increasing the temperature vibration frequency, more pyroelectric charges were produced to increase the catalytic efficiency. Consequently, without any sacrificial reagents, the designed system is effective for the pyroelectric catalytic splitting of water, with a H_2_ production rate as high as 0.654 μmol h^−1^. This study provided new insights into making full use of sunlight‐driven temperature vibration energy in a pyroelectric catalytic process.^[37]^

**Figure 7:**
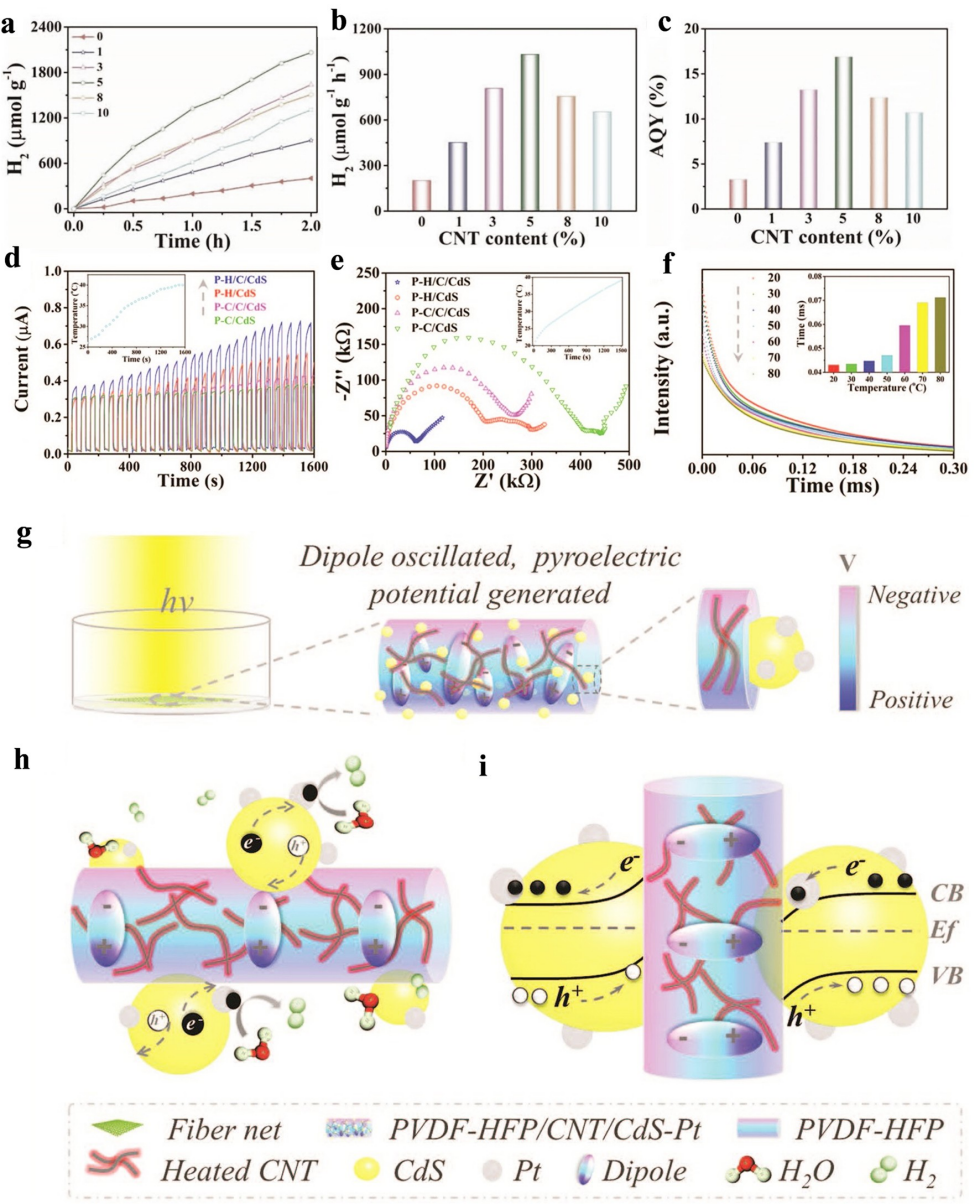
Evaluation of the rate of hydrogen production and AQY of composite microfibers. a) Rates of hydrogen evolution using PVDF‐HFP/CNT/CdS−Pt fibers with different CNT contents. Comparison of the hydrogen generation rates (b) and AQY (c) of the above fibers. Characterization of the photogenerated carrier behavior of the samples PVDF‐HFP/CNT/CdS (P−H/C/CdS), PVDF‐HFP/CdS (P−H/CdS), PVDF‐CTFE/CNT/CdS (P−C/C/CdS), and PVDF‐CTFE/CdS (P−C/CdS). d) Photocurrent outputs, e) electrochemical impedance spectroscopy (EIS) Nyquist plots, f) the relative temperature‐dependent photoluminescence (PL) intensity (integrated from 425 to 525 nm, ex. 325 nm). Schematic diagram of IR‐responsive photoinduced carriers for the enhanced photocatalytic generation of hydrogen. g) Schematic representation of the photocatalytic composite microfiber PVDF‐HFP/CNT/CdS−Pt semi‐immersed in water. Under irradiation with light, the CNT absorbs infrared light and is heated first, which then heats the microfiber substrate PVDF‐HFP. The dipoles of the pyroelectric substrate PVDF‐HFP oscillate more evidently and, thus, a pyroelectric potential forms on the microfiber surface. h) Photoexcited carriers from CdS move in opposite directions, driven by the pyroelectric field. Electrons migrate to the co‐catalyst Pt and react with water molecules to generate hydrogen. i) Energy band bending for the ideal interface between the microfiber substrate PVDF‐HFP and CdS. Reproduced with permission.^[38]^ Copyright 2020, Wiley‐VCH.

In addition, the pyroelectric process can also be coupled with photocatalysis to promote the migration/separation of charge carriers, thus increasing the catalytic activity as a result of the presence of a built‐in electric field. For example, Zhao and co‐workers recently designed a novel infrared‐light‐responsive photocatalytic composite microfiber (PVDF‐HFP/CNT/CdS). The composite consisted of vinylidene fluoride‐*co*‐hexafluoropropylene (PVDF‐HFP), carbon nanotubes (CNTs), and CdS, in which the CdS served as a typical photocatalyst for water splitting, the CNTs acted as photothermal materials that converted the solar energy to heat, thus increasing the system temperature, and the PVDF‐HFP functioned as a piezo‐polymer with a pyroelectric effect. Under irradiation with light, the CNTs harvested IR light and converted it into heat to induce a temperature increase (d*T*/d*t*>0), thereby leading to the production of pyroelectric positive and negative charges on opposite surfaces of PVDF‐HFP with a built‐in electric field. The built‐in electric field can make great contributions to promoting the migration and separation of charge carriers, as demonstrated by the transient photocurrent, electrochemical impedance spectroscopy, and time‐resolved photoluminescence. Consequently, compared with PVDF‐HFP/CdS, the photocatalytic H_2_ production rate of the PVDF‐HFP/CdS/CNT was improved 5.3 times, with a highest apparent quantum yield (AQY) of up to 16.9 %. This study provided a unique and efficient way for establishing coupled photocatalytic systems with optimized activity for water splitting (Figure 7a–i).^[38]^ Although, all five types of reactions mentioned above have different reaction mechanisms, with different energy conversion pathways, their ultimate outcomes are all chemical energy. Furthermore, sunlight energy is applied as the energy source in all the above catalytic reactions. Therefore, to avoid confusion, in this Minireview we have given them a specific and “all‐in‐one” definition, namely, “Solar Energy Catalysis” (SEC). Table 1, summarizes different types of SEC reactions.

**Table 1:** Different types of catalytic reactions driven by solar energy.

Catalysts		Catalytic condition		Catalytic applications		Types of SEC reaction		Catalytic activity		Ref.
Pt−Co_3_O_4_/18‐facet SrTiO_3_		UV light (300 W Xe lamp)		Overall water splitting		Photocatalysis		AQE value is 0.81 % at 365 nm		[15]
Ta_3_N_5_/Rh/Cr_2_O_3_		Visible light (300 W Xe lamp) or solar simulator AM 1.5G		Overall water splitting		Photocatalysis		AQE values are 2.2 % at 320 nm, 0.22 % at 420 nm, 0.024 % at 500 nm		[16a]
Bi_4_NbO_8_Cl/Rh‐doped SrTiO_3_		300 W Xe lamp fitted with L‐42 cut off filter		Overall water splitting		Photocatalysis		About 304.7 μmol of O_2_ for 60 h		[17a]
Pt‐loaded MgTa_2_O_6−*x*_N_y_/TaON/PtO_*x*_−WO_3_		Full light (300 W xenon lamp)		Overall water splitting		Photocatalysis		AQE value is 6.8 % at 420 nm		[17b]
COF‐318‐TiO_2_		A xenon arc lamp (200 mW cm^−2^) with a light filter (380–800 nm)		CO_2_ reduction		Photocatalysis		CO_2_‐to‐CO conversion efficiency of 69.67μmol g^−1^/h		[8d]
3DOM CdSQD/NC		300 W xenon lamp with a 420 nm cut‐off filter		CO_2_ reduction		Photocatalysis		AQE value is 2.9 % at 450 nm		[8e]
Single‐atom Ni−OB−CN photocatalyst		300 W Xe lamp		CO_2_ reduction		Photocatalysis		CO and CH_4_ production rates of 22.1 and 8.7 μmol g^−1^ h^−1^		[8f]
TPA‐PQ		A 300 W xenon arc lamp with a visible band pass filter (λ>420 nm)		CO_2_ reduction		Photocatalysis		CO_2_ to CH_4_ yield of 32.2 mmol g^−1^, with a rate of 2.15 mmol h^−1^ g^−1^ and high selectivity of 97 %		[8g]
Er^3+^−TiO_2_		UV/Vis‐NIR light		Phenol degradation		Up‐conversion effect promoted photocatalysis		About 90 % phenol is degraded after 120 min		[18a]
QDs/g‐C_3_N_4_		Visible light (300 W xenon lamp)		Organic dye degradation		Up‐conversion effect promoted photocatalysis		54 % MO (*k*a=0.0141) is degraded after 60 min		1[8b]
C−TiO_2_/three color emitting up‐conversion phosphors		LEDs and NIR laser		NO*x* decomposition		Up‐conversion effect promoted photocatalysis		NIR light induced photocatalytic ability with about 9.3 % of NO destruction		[18c]
(UCNPs)‐Pt@MOF/Au		Full solar light equipped with AM 1.5G		H_2_ evolution		Up‐conversion effect promoted photocatalysis		H_2_ evolution rate of 280 μmol g^−1^ h^−1^		[18d]
Co‐*x* catalysts		UV/Vis irradiation (300 W Xe light)		Fischer–Tropsch synthesis		Light‐driven thermal catalysis		An olefin (C_2–4_^=^) selectivity of 36.0 % and an olefin/paraffin ratio of 6.1 at a CO conversion of 15.4 %		[22]
MnO‐modified Ni‐based catalyst systems		UV/Vis irradiation (300 W Xe light)		CO hydrogenation to light olefins		Light‐driven thermal catalysis		33 % selectivity to light olefins at a CO conversion of 14.9 %		[23]
Metal‐organic‐framework derived hollow N‐doped porous carbon		300 mW cm^−2^ full spectrum irradiation		CO_2_ cycloaddition		Light‐driven thermal catalysis		The yield of cycloaddition of CO_2_ with 3‐bromopropylene oxide is 94 %		[24]
PMo_12_@Zr−Fc MOFs		Light irradiation with sunlight intensity of 0.2 W cm^−2^		CO_2_ cycloaddition		Light‐driven thermal catalysis		The yield of cycloaddition of CO_2_ with styrene oxide is 88.05 %		[3i]
Zn SA−NC		300 mW cm^−2^ full spectrum irradiation		CO_2_ cycloaddition		Light‐driven thermal catalysis		Yields of 99 % for epichlorohydrin and 97 % for 3‐bromopropylene oxide		[3f]
Ni@C−X		UV/Vis‐IR light irradiation		CO_2_ reduction		Light‐driven thermal catalysis		CH_4_ production rates of 488 mmol g^−1^ h^−1^		[3a]
Plasmonic ZnCu alloy catalyst		AM 1.5 light as the illuminant, and a maximum light intensity of 788 mW cm^−2^		Water and methanol activation for H_2_ production		Light‐driven thermal catalysis		H_2_ production rate of 328 mmol g^−1^ h^−1^ with a solar energy conversion efficiency of 1.2 %		[3b]
Cu@HKUST‐1 composites		300 W Xe lamp		Cascade reactions (hydrogenation of nitrobenzene followed by reductive amination of benzaldehyde)		Light‐driven thermal catalysis		100 % selectivity toward N‐benzylideneaniline over 10 h		[3c]
Pd nanocubes@ZIF‐8		60 mW cm^−2^ full‐spectrum		Hydrogenation of 1‐hexene		Light‐driven thermal catalysis		Approximately 100 % conversion in 90 mins		[3e]
Ga−Cu/CeO_2_		300 W xenon arc lamp was utilized as the illuminant		CO_2_ reduction		Light‐driven thermal catalysis		A CO production rate of 111.2 mmol g^−1^ h^−1^ with nearly 100 % selectivity		[3j]
Rh/Al nanoantenna catalyst		Simulated solar irradiation (11.3 W ⋅ cm^−2^)		CO_2_ methanation		Light‐driven thermal catalysis		CH_4_ productivity of 550 mmol g^−1^ h^−1^ with nearly 100 % selectivity		[3g]
Ag@SiO_2_@TiO_2_−Au		UV and full‐spectrum irradiation		H_2_ evolution		Thermal‐assisted photocatalysis		H_2_ evolution rate of 30.2 mmol g^−1^ h^−1^		[26]
Bilayer paper from commercialized TiO_2_ and carbon nanomaterials		Full‐spectrum irradiation		Photocatalytic phenol oxidation		Thermal‐assisted photocatalysis		88.4 %mineralization of high concentration phenol within 90 min		[27]
Silver‐decorated titanium oxide at a gas–water boundary		Full‐spectrum irradiation		CO_2_ reduction		Thermal‐assisted photocatalysis		CO_2_ reduction rate of 305.7 μmol g^−1^ h^−1^		[28]
Pt/PCN‐224(M)		Visible light irradiation with relatively low intensity (<100 mW cm^−2^)		Oxidation of primary alcohols to aldehydes		Thermal‐assisted photocatalysis		The yield of oxidation of benzyl alcohol by O_2_ is >99 % within 50 min		[25a]
Triphenylbenzene‐dimethoxyterephthaldehyde‐COF		Visible light (300 W Xe lamp)		Photocatalytic hydrogen peroxide production		Thermal‐assisted photocatalysis		H_2_O_2_ production rate of 2.9 mmol g^−1^ h^−1^		[25b]
Ni/mesoporous TiO_2_		Xenon light (XE‐300F) is used as the light source		Methane dry reforming reaction		Thermal‐assisted photocatalysis		The average conversions of CH_4_/CO_2_ increased by around 14.18 % and 15.91 %		[25c]
Ni/TiO_2_		Solar light simulator (CEL‐HXF300, 300 W Xenon lamp)		N_2_ hydrogenation		Thermal‐assisted photocatalysis		Ammonia yield of 19.9 μg (g_cat_h)^M‐>1^		[25d]
Ru@Ni_2_V_2_O_7_		300 W Xe lamp irradiation (about 2.0 W cm^−2^)		Sabatier reaction		Photothermal synergistic catalysis		CO_2_ methanation rate of 114.9 mmol g^−1^ h^−1^		[30]
Pd_3_Cu@UiO‐66		Light irradiation with a 300 W Xe lamp		CO_2_ hydrogenation		Photothermal synergistic catalysis		A methanation production rate of 340 μmol g^−1^ h^−1^		[31]
M‐doped TiO_2_ (M=Zn, Ni, and Cu)		300 W Xe lamp		CO_2_ conversion		Photothermal synergistic catalysis		Stable production of CO of 10.80 μmol g^−1^		[29f]
Fe‐based catalysts		Light irradiation		CO_2_ conversion		Photothermal synergistic catalysis		11.3 mmol g^−1^ h^−1^ activity for the catalytic conversion of CO_2_		[29c]
NiFe DLH/Ni foam coupled with/perovskite tandem cell		Simulated AM 1.5G solar irradiation (100 mW cm^−2^)		Overall water splitting		Solar cell powered electrocatalysis		Solar‐to‐hydrogen efficiency of 12.3 %		[33]
NiMo alloy NiFe alloy/perovskite/Si tandem semiconductors		incident irradiation power (100 mW m^−2^)		Overall water splitting		Solar cell powered electrocatalysis		Solar‐to‐hydrogen efficiency of 20 %		[34]
Perovskite‐organic monolithic tandem solar cells/NiFe LDH electrodes		Simulated AM 1.5G illumination		Overall water splitting		Solar cell powered electrocatalysis		Solar‐to‐hydrogen efficiency of 12.30 % and 11.21 % for rigid, and flexible perovskite‐organic tandem solar cell based PV‐driven electrolysis systems		[32b]
A p^+^nn^+^‐Si/Ti/Pt photocathode		Simulated AM 1.5G illumination		Overall water splitting		Solar cell powered electrocatalysis		17.6 % STH efficiency		[5a]
two monolithically encapsulated perovskite solar cells with CoP and FeNi(OH)*x* co‐catalysts		Simulated AM 1.5G illumination		Overall water splitting		Solar cell powered electrocatalysis		Solar‐to‐hydrogen efficiency of 8.54 %		[5b]
NiCoFe‐based electrocatalyst/monolithic perovskite/silicon tandem solar cell		Simulated AM 1.5G illumination		Overall water splitting		Solar cell powered electrocatalysis		20 % solar‐to‐hydrogen efficiency		[5f]
SnO_2_ modified CuO nanowire electrodes/GaInP/GaInAs/Ge photovoltaic		Simulated AM1.5G spectrum at 1 sun intensity		CO_2_ reduction		Solar cell powered electrocatalysis		A solar‐to‐CO efficiency of 13.4 %		[35]
CuAg bimetallic cathode/four‐terminal III–V S^−1^i tandem solar cell		1 Sun illumination		CO_2_ reduction		Solar cell powered electrocatalysis		Conversion efficiency to hydrocarbons and oxygenates exceeding 5 %		[36]
GaAs solar cell/nano‐Au electrocatalyst		1 Sun illumination		CO_2_ reduction		Solar cell powered electrocatalysis		Solar‐to‐CO photoconversion efficiency of 15.6 %		[5d]
Si solar cell/Cu catalyst		1 Sun illumination		CO_2_ reduction		Solar cell powered electrocatalysis		Solar conversion efficiencies of 4.47 % and 6.4 % for C_2_H_4_ and C_2+_		[5c]
Lead zirconate titanate		Thermal cycling		H_2_ evolution		Pyroelectric catalysis		H_2_ evolution rate of 0.654 μmol/h		[37]
PVDFHFP/CNT/CdS		280 W xenon lamp with simulated full‐spectra solar light		H_2_ evolution		Pyroelectric catalysis		Apparent quantum yield of 16.9 %		[38]

## 3 Summary and Outlook

In summary, utilizing light energy to drive catalytic reactions is considered a green and sustainable technology for resolving the problems of energy shortage and environmental pollution, and various technologies have been developed to make full use of sunlight. To avoid confusion, we have used “Solar Energy Catalysis” (SEC) as a specific and “all‐in‐one” definition for catalytic reactions that utilize sunlight as the energy source. Through different energy conversion pathways, solar energy can be ultimately converted into chemical energy through SEC processes. To provide an in‐depth understanding, the various types of SEC processes, including photocatalysis (up‐conversion effect promoted photocatalysis), photothermal catalysis (light‐driven thermal catalysis, thermal‐assisted photocatalysis, and photothermal synergistic catalysis), solar cell powered electrocatalysis, and pyroelectric catalysis, have been systematically introduced. The reaction mechanisms of different SEC processes have also been discussed from the perspective of the different components of the catalytic systems and energy conversion pathways. Moreover, the similarities and differences between different SEC processes have also been highlighted. As a promising research topic, SEC processes have great potential for triggering different kinds of catalytic reactions with solar energy as the input energy source, such as water splitting, CO_2_ reduction, cycloaddition, as well as nitrogen fixation. Therefore, we want to share our insights into the opportunities and challenges for the further development of SEC reactions.

### 3.1 Developing New Materials

At present, various materials have been studied in the field of SEC reactions, especially oxides, sulfides, and phosphides. However, the most widely studied materials always have a rigid structure, which makes it hard to regulate their properties in a controllable manner, thus restricting their catalytic activity improvement. Therefore, developing new materials, such as metal‐organic frameworks (MOFs),^[39]^ covalent organic frameworks (COFs),^[40]^ and 2D materials,^[41]^ is urgently required. MOFs and COFs display many advantages in SEC reactions: 1) they possess porous frameworks, which provide more reactive sites and are beneficial for mass transfer; 2) their structures can be easily tailored to regulate their properties by changing the building units; and 3) they have a distinct structure, which provides an ideal platform for understanding their catalytic mechanism. 2D materials are another kind of emerging catalysts, which show great potential in SEC reactions because of their ultrathin thickness and, thus, more abundant active surface atoms, reduced charge transfer pathways, and tailored band structures.

### 3.2 Modulating the Nanostructure of SEC Catalysts

The nanostructure of materials, such as their dominant crystal facet, size, morphology, and porous structure, has a great impact on their catalytic activity, since the nanostructure of a material is closely related to its light absorption, band structure, distribution of reactive sites, mass transfer, as well as thermal generation performance. For example, different crystal facets of the same materials display different atomic arrangements on the exposed facets, and possess different amounts of surface reactive sites, which results in different SEC activities. Moreover, further reducing the size of the materials will lead to quantum confinement effects occurring, which will widen the band gap and enhance their redox ability. Therefore, carefully modulating their nanostructure is an efficient method to optimize the SEC activity of catalysts.

### 3.3 In‐Depth Understanding of the Different SEC Reaction Mechanisms

Although great achievements have been made in the field of solar energy catalysis in recent years, the reaction mechanisms of some SEC reactions are still ambiguous, which should be further explored. For example, pyroelectric catalysis is an emerging class of SEC reactions, and currently mechanistic studies on them are ambiguous and not comprehensive. Some reported studies explain the mechanism from the perspective of semiconductor catalysis, in which the active substances are the electron–hole pairs generated by temperature fluctuation. In contrast, some researchers believe that the mechanism of pyroelectric catalysis should be similar to that of electrocatalysis. Moreover, the energy transfer pathway for photothermal catalysis is diverse and complex, with the main energy transfer pathway often being from light to heat energy. In contrast, however, some systems are dominated by the transfer of energy from light to electronic excited states. In addition to energy transfer pathways, the defects, atomic arrangement, and molecular adsorption properties on the surface of the SEC catalysts also needs to be more deeply investigated, which would be beneficial for understanding the relationship between the structure of the catalysts and their catalytic performance. Various characterization methods have been developed to achieve this goal, but most of them can only indicate the properties of the catalysts before and after the reaction. However, information on the properties of the catalysts during the reaction—such as changes in their atomic structure, intermediate state of the reactants, reduction and oxidation state of their surface reactive sites, as well as coordination atom migration—are normally transient and is more important for us to obtain an in‐depth understanding of the different mechanisms of SEC reactions. Therefore, in situ characterization techniques are in great need to explore the relationship in depth between the structure of catalysts and their catalytic performance at the nanoscale or even the atomic level. It is promising that different kinds of in situ characterization methods have been successfully designed and used for studying the mechanism of the catalytic reactions. For example, in situ XPS, XRD, and EXAFS can help us investigate microstructural variations of the catalysts during the catalytic reactions, in situ DRIFTS can help us determine the intermediate state of the reactants, and in situ EXAFS and STEM can provide information about the process of catalyst formation. Thus, combining advanced in situ characterization and theoretical calculation methods may provide an effective way to obtain an in‐depth understanding of catalytic mechanisms.

### 3.4 Designing More Efficient Devices

Although it is very important for us to understand the reaction mechanisms and explore more active materials to improve the conversion efficiency of SEC processes, a more difficult challenge for researchers is to achieve the goal of mass producing the catalysts and assembling them into devices for industrialization. In the field of photocatalysis, in particular, most investigations remain at the laboratory stage because of poor light conversion efficiency of the catalysts, problems in recycling powder catalysts, high cost, and low stability. Constructing devices may not only contribute to recycling the catalysts, but their light absorption and electron–hole pairs separation performance could also be enhanced through coupling with other catalytic technologies, such as thermal catalysis, pyroelectric catalysis, and electrocatalysis. In this regard, the Domen group made some outstanding studies in recent years. For example, by using Al‐doped SrTiO_3_ particles as photocatalysts, they successfully arranged 1600 device units to build a 100 m^2^ scale prototype photocatalytic solar hydrogen production system.^[42]^ Although, the STH values still need to be improved, this study provides hope that photocatalytic H_2_ production could have a practical application. We hope this Minireview will provide researchers with a new insight into the SEC process and be beneficial for future research in this field.

## Conflict of interest

The authors declare no conflict of interest.

## Biographical Information

*Xiaodong Sun received his Ph.D. in inorganic chemistry from Jilin University in 2019. Currently, he is an assistant research associate at Liaoning University. His current research concentrates on the design and synthesis of MOF‐based photocatalysts for energy generation*.

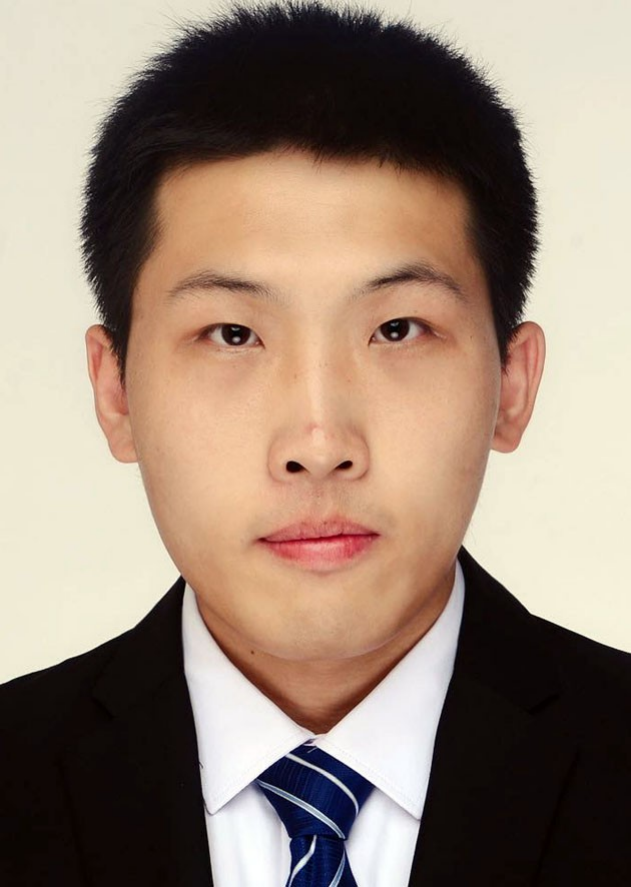

## Biographical Information

*Hongwei Huang is a Professor at Beijing Key Laboratory of Materials Utilization of Nonmetallic Minerals and Solid Wastes, School of Materials Science and Technology, China University of Geosciences (Beijing). He received his Ph.D. in 2012 from the Technical Institute of Physics and Chemistry, Chinese Academy of Sciences, and worked as a visiting scholar with Prof. Thomas Mallouk at The Pennsylvania State University (2016–2017). His current research mainly focuses on crystal structural design and charge regulation of polar photocatalytic nanomaterials and their application for solving environmental and energy issues*.

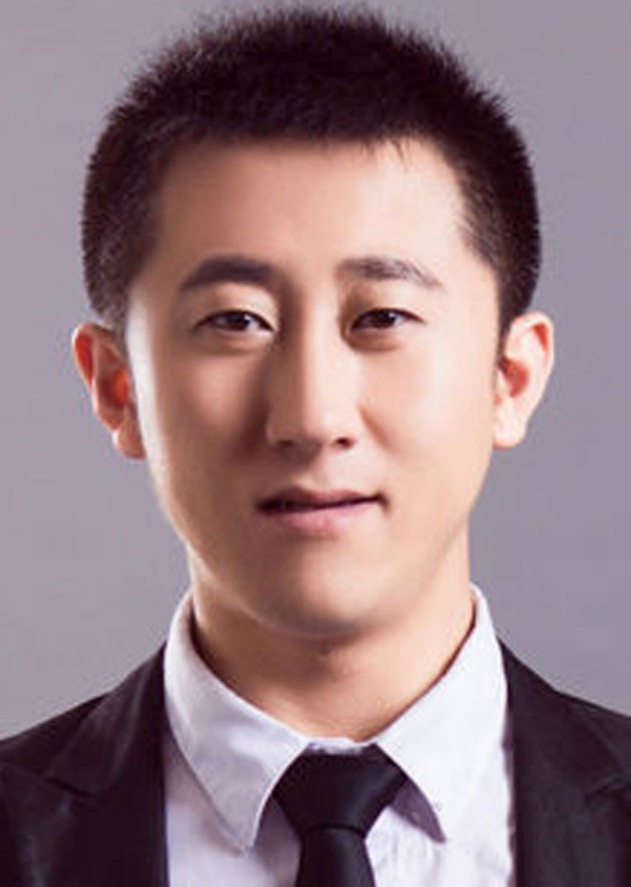

## Biographical Information

*Prof. Tianyi Ma received his PhD in Physical Chemistry in 2013 from Nankai University, China. He is a Fellow of the Royal Society of Chemistry and Clarivate's Global Highly Cited Researcher. He was awarded an Australian Research Council (ARC) Discovery Early Career Researcher Award (DECRA) in 201, and an ARC Future Fellowship in 2021. He is currently a full professor at RMIT University, where he focuses on energy materials and catalysis for renewable energy conversion and storage as well as carbon capture, usage, and storage (CCUS)*.

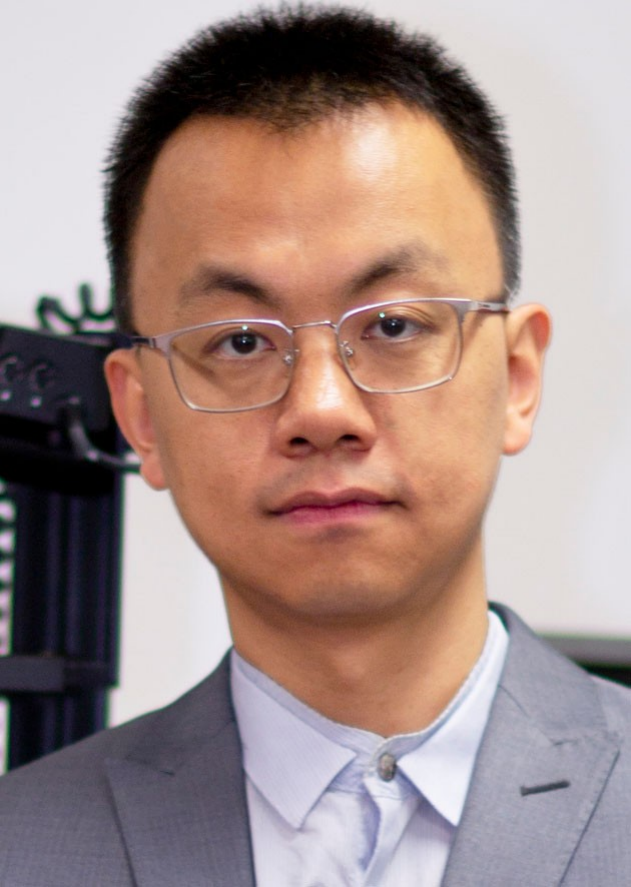

## Biographical Information

*Professor Baohua Jia is the Founding Director of Centre for Atomaterials Science and Technology (CAST) at RMIT University. She is a Fellow of Optical Society and ARC Future Fellow. She received her BSc and MSc degrees from Nankai University, China. She was awarded a PhD (2007) from Swinburne University of Technology, Australia. Dr Jia's research focuses on the fundamental light and nano‐and atomaterial interaction. In particular her work on laser manipulation of two‐dimensional materials has led to the design and fabrication of functional nanostructures and nanomaterials for effective harnessing and storage of clean energy from sunlight, purifying water and air for clean environment and imaging and spectroscopy and nanofabrication using ultrafast laser towards fast‐speed all‐optical communications and intelligent manufacturing*.